# Acute and Repeated Ashwagandha Supplementation Improves Markers of Cognitive Function and Mood

**DOI:** 10.3390/nu16121813

**Published:** 2024-06-08

**Authors:** Megan Leonard, Broderick Dickerson, Landry Estes, Drew E. Gonzalez, Victoria Jenkins, Sarah Johnson, Dante Xing, Choongsung Yoo, Joungbo Ko, Martin Purpura, Ralf Jäger, Mark Faries, Wesley Kephart, Ryan Sowinski, Christopher J. Rasmussen, Richard B. Kreider

**Affiliations:** 1 Exercise & Sport Nutrition Lab, Department of Kinesiology and Sports Management, Texas A&M University, College Station, TX 77843, USA; meganleonard10@tamu.edu (M.L.); dickersobl5@email.tamu.edu (B.D.); landry.estes@tamu.edu (L.E.); dg18@tamu.edu (D.E.G.); victoria.jenkins@tamu.edu (V.J.); sjohnson2216@tamu.edu (S.J.); dantexing@tamu.edu (D.X.); choongsungyoo@tamu.edu (C.Y.); joungboko10@tamu.edu (J.K.); mark.faries@ag.tamu.edu (M.F.); rjs370@tamu.edu (R.S.); crasmussen@tamu.edu (C.J.R.); 2 Increnovo LLC, Whitefish Bay, WI 53217, USA; martin.purpura@increnovo.com (M.P.); ralf.jaeger@increnovo.com (R.J.); 3 Texas A&M AgriLife Extension, Texas A&M University, College Station, TX 77843, USA; 4 Department of Kinesiology, University of Wisconsin—Whitewater, Whitewater, WI 53190, USA; kephartw@uww.edu

**Keywords:** nootropic, executive function, cognition, memory, vigilance, attention, mood

## Abstract

Background: Ashwagandha has been reported to reduce stress and attenuate cognitive decline associated with inflammation and neurodegeneration in clinical populations. However, the effects as a potential nootropic nutrient in younger populations are unclear. This study examined the effects of liposomal ashwagandha supplementation on cognitive function, mood, and markers of health and safety in healthy young men and women. Methods: 59 men and women (22.7 ± 7 yrs., 74.9 ± 16 kg, 26.2 ± 5 BMI) fasted for 12 h, donated a fasting blood sample, and were administered the COMPASS cognitive function test battery (Word Recall, Word recognition, Choice Reaction Time Task, Picture Recognition, Digit Vigilance Task, Corsi Block test, Stroop test) and profile of mood states (POMS). In a randomized and double-blind manner, participants were administered 225 mg of a placebo (Gum Arabic) or ashwagandha (*Withania somnifera*) root and leaf extract coated with a liposomal covering. After 60-min, participants repeated cognitive assessments. Participants continued supplementation (225 mg/d) for 30 days and then returned to the lab to repeat the experiment. Data were analyzed using a general linear model (GLM) univariate analysis with repeated measures and pairwise comparisons of mean changes from baseline with 95% confidence intervals (CI). Results: Ashwagandha supplementation improved acute and/or 30-day measures of Word Recall (correct and recalled attempts), Choice Reaction Time (targets identified), Picture Recognition (“yes” correct responses, correct and overall reaction time), Digit Vigilance (correct reaction time), Stroop Color-Word (congruent words identified, reaction time), and POMS (tension and fatigue) from baseline more consistently with several differences observed between groups. Conclusion: Results support contentions that ashwagandha supplementation (225 mg) may improve some measures of memory, attention, vigilance, attention, and executive function while decreasing perceptions of tension and fatigue in younger healthy individuals. Retrospectively registered clinical trial ISRCTN58680760.

## 1. Introduction

Ashwagandha (*Withania somnifera*) is a plant used in Ayurvedic medicine for over 3000 years as a naturally occurring adaptogen to help manage stress, anxiety, and inflammation [1,2,3,4,5,6,7,8,9,10,11,12,13]. Ashwagandha has antioxidant properties [9,14,15,16], influences endocrine function [17,18,19,20,21], and has immunomodulatory effects [10,22]. Ashwagandha supplementation has been reported to attenuate cognitive decline associated with inflammation [9,11,12] and neurodegeneration [8,10,15,23,24,25,26]. Since oral ingestion of ashwagandha is bioavailable and crosses the blood–brain barrier [24], ashwagandha has been considered a naturally occurring therapeutic agent for individuals with type 2 diabetes mellitus [9,10,25], mild cognitive impairment [8,11,14] and individuals with neurodegenerative diseases [14,15,24,27,28,29].

Basic research studies provide a solid theoretical rationale that ashwagandha supplementation may benefit health and cognition [25,27,30]. However, fewer studies have evaluated the effects of ashwagandha supplementation on cognitive function in clinical and healthy populations [31]. For example, Chengappa et al. [32] reported that ashwagandha supplementation (2 × 250 mg/d for eight weeks) in 60 patients with medically managed bipolar disorders improved measures of working memory, reaction time, and social cognition. Choudhary and associates [33] reported that ashwagandha supplementation (2 × 300 mg/d for eight weeks) in 50 individuals with mild cognitive impairment (MCI) improved measures of immediate and general memory, executive function, attention, and information processing speed. This group also reported that ashwagandha supplementation (2 × 300 mg/d for eight weeks) reduced cortisol and improved mental well-being in 52 patients suffering from chronic stress and related disorders [7]. Similarly, Remenapp et al. [34] reported that supplementation with a liposomal-coated ashwagandha (225 or 400 mg/d for 30 days) reduced markers of stress and improved cognitive function in individuals experiencing perceived stress. In healthy individuals, Pingali and collaborators [35] reported that ashwagandha supplementation (2 × 250 mg/d for 14 days) improved reaction times and cognitive and psychomotor performance in 20 healthy young males. Moreover, Baker and coworkers [36] reported that ashwagandha supplementation (2 × 350 mg/d for 30 days) improved college students’ perceptions of well-being, energy, mental clarity, and sleep quality. Our group found that acute ingestion of ashwagandha (400 mg) enhanced measures of executive function, short-term/working memory, and the ability to sustain attention for up to six hours in healthy individuals [37]. Additionally, Bonilla and coworkers [38] conducted a Bayesian meta-analysis and systemic literature review and found evidence that ashwagandha supplementation improved physical performance-related variables. While these findings are promising, more research is needed to determine the acute and long-term effects of ashwagandha supplementation on cognitive function and health measures, particularly in healthy populations.

This study aimed to examine the effects of liposomal ashwagandha supplementation (acute dose and 225 mg/d for 30 days) on a comprehensive battery of cognitive function assessments, mood, and markers of health and safety in healthy young men and women. The primary outcomes were cognitive function and measures. Secondary outcomes included markers of health and safety. We hypothesized that acute supplementation with ashwagandha would increase markers of cognitive function and that 30-day supplementation of ashwagandha would result in additive benefits while well tolerated. The following describes the study’s methods, procedures, and results, followed by a discussion and recommendations for additional work.

## 2. Methods

### 2.1. Experimental Design of Study

This clinical trial was conducted at a university clinical research facility in a double-blind, placebo-controlled manner. The independent variable was nutritional supplementation. Primary dependent variable outcomes included measures of cognitive function. Secondary outcomes were markers of health obtained from clinical chemistry panels and perceptions of side effects.

### 2.2. Participants of the Study

This clinical trial was retrospectively registered with the ISRCTN registry on 15 May 2024 (ISRCTN58680760). The study was approved by the Human Research Protection Program Review Board (IRB2022-0621, 21 September 2022) and adhered to the Declaration of Helsinki ethical standards for conducting human participant research. Participants were recruited for this study through emails and advertising on flyers, websites, and online flyers. Potential subjects were screened via questionnaires to determine initial eligibility and invited to attend a familiarization session if they appeared to meet eligibility criteria. Those interested in participating in the study signed consent statements, completed health history questionnaires, and underwent physical examination to confirm eligibility.

Healthy volunteers between 18 and 60 years of age were recruited for this study with the following inclusion criteria: They must (1) have no diagnosed cognitive deficits from a physician; (2) have no diagnosed sleep disorders from a physician; (3) have no history of cardiovascular, metabolic or pulmonary disease from a physician; (4) have no history of migraine headaches, hypertension, cardiac arrhythmias or anxiety; (5) have no ulcers or gastrointestinal reflux disease; (6) be willing to provide voluntary, written, and informed consent; (7) be willing to consume the investigational product daily for the duration of the study; and (8) have no allergies to the fiber Gum Arabic. Participants were excluded from the study if they (1) were pregnant or desired pregnancy during the study; (2) had a documented history of taking prescription medications in the prior month that might affect study testing. Individuals taking medications that the investigators deemed would not affect primary study outcomes and were taken throughout the study (e.g., glucose management, lipid-lowering, anti-hypertensive, thyroid medications, etc.) were permitted to participate in the study; and (3) were recently instructed by their physician (within the past month) to abstain or limit caffeine or other products containing stimulants.

**Figure 1:**
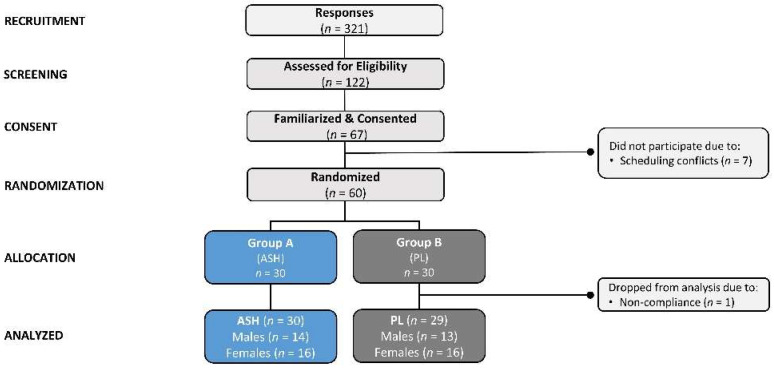
Consolidated Standards of Reporting Trials (CONSORT) flow chart for the ashwagandha (ASH) and placebo (PL) groups.

A Consolidated Standards of Reporting Trials (CONSORT) illustration is provided in Figure 1. A total of 321 individuals underwent phone screening to assess general eligibility. Of these, 122 cleared the initial screening criteria and were invited to familiarization sessions. Sixty-seven people were familiarized with the study and provided informed consent. Of these, 7 subjects were unable to participate in the study due to scheduling conflicts, and 60 individuals were enrolled in the study and matched according to age, body mass index, and sex for random assignment into treatment groups. A total of 30 participants were randomized into the placebo (PLA) group and 30 into the ashwagandha (ASH) group. One was removed from the study due to non-compliance. Data from 59 participants were analyzed statistically.

### 2.3. Testing Sequence

**Figure 2:**
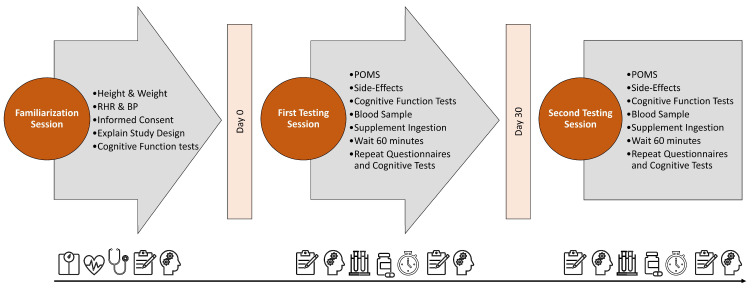
Testing timeline. RHR indicates resting heart rate, BP indicates resting blood pressure, and POMS represents Profile of Mood States inventory.

Figure 2 shows the experimental timeline. Participants attended one familiarization and two experimental testing sessions. At the familiarization session, participants completed health history questionnaires and had height, weight, and resting hemodynamics determined. The methods and expectations of the study were also described. Those eligible to participate practiced the cognitive function tests at least three times to familiarize themselves with the assessments and minimize the learning effects. Participants were instructed on recording diet and energy-containing beverage intake and provided a list of foods and beverages containing stimulants to avoid consuming before each testing session.

Before the first testing session, participants recorded their food and beverage intake for 4 days. They were also asked to refrain from consuming unusual amounts of caffeine and other stimulants for 48 h, fast for 12 h, and refrain from intense exercise for 24 h before testing. Upon reporting to the lab, participants completed a pre-supplementation (Pre) side-effect questionnaire and performed Computerized Mental Performance Assessment System (COMPASS) cognitive tests, including Word Recall, Word Recognition, Choice Reaction Time, Picture Recognition, Corsi Block, Digit Vigilance, and Stroop Color–Word test) as well as a Profile of Mood States (POMS) questionnaires. Participants then donated a fasted blood sample. Participants were randomly assigned to a placebo or ashwagandha supplement. One hour following ingestion, participants repeated cognitive testing through COMPASS, the POMS questionnaire, and a post-supplement side effect questionnaire. Participants were then given a 30-day supply of the supplement ingested during the visit and, in between testing sessions, were instructed to ingest daily in the morning with breakfast. Participants maintain their regular diet and physical activity levels and return to the lab after 30 days of supplementation to repeat the testing protocol from the first session and ingest the last dose of their randomly assigned supplement.

### 2.4. Supplementation Protocol

Participants consumed one capsule per day of either a placebo (PL) consisting of 225 mg of Gum Arabic (Spraygum Bai, Lot #190 057, Nexira Food, Rouen, France) or 225 mg of liposomal ashwagandha (*Withania somnifera*) root and leaf extract (ASH, NooGandha^®^, Specnova LLC, Lot #221221, Tysons Corner, VA, USA). This dosage and source of ashwagandha had been previously shown to reduce markers of stress and improve cognitive function in individuals with perceived stress [34]. The liposomal root and leaf extract was manufactured using sunflower lecithin consisting of a proprietary blend of phosphatidylcholine, phosphatidylserine, phosphatidylinositol, phosphatidylethanolamine, and phosphatidic acid, and a surface coating using gum Arabic-derived polysaccharides and ashwagandha plant fibers to improve the stability of the liposomes as they pass through the gastrointestinal tract. A homogenizer was used to mix the liposomal formulation with the ashwagandha extract in water/ethanol and then spray dried. The raw ingredients were encapsulated using Vcaps^®^ Plus Capsules (Capsugel^®^, Lot #5412055, Colmer, France). Certificates of analysis were provided by the raw ingredient supplier and the company that encapsulated the supplements, verifying dosage and ensuring that the supplements were free from contaminants. Capsules were the same size and color and were shipped in labeled bags. Once received, the capsules were placed into individual participant supplementation containers and labeled as designated for double-blinded administration. Participants ingested the supplements after breakfast (or about 8:00 a.m.) for 29 days. Participants ingested the supplement on day 30 following pre-supplementation cognitive testing and obtaining a blood sample.

## 3. Procedures

### 3.1. Participant Descriptives

Weight and height were obtained from a Health-O-Meter Professional 500KL (Pelstar LLC, Alsip, IL, USA) digital scale. After sitting passively for 5 min, resting heart rate and blood pressure were obtained using standard procedures with a Connex^®^ ProBP™ 3400 monitor (Welch Allyn, Tilburg, The Netherlands).

### 3.2. Diet Control

Volunteers documented beverage and food consumption for four days before baseline assessments using written food logs or a phone application (MyFitnessPal, Inc., Baltimore, MD, USA) [37]. This initial diet was replicated before the second testing session for diet consistency.

### 3.3. Cognitive Function Assessment

The Computerized Mental Performance Assessment (COMPASS) software version 6.0 (Northumbria University, Newcastle, UK) was used to evaluate measures of cognitive function. The assessments included (1) the Word Recall test that measures working memory and the transfer to long-term memory through secondary memory by recalling words shown 60 seconds (immediately) [39] after being seen and recalling them again at the end of cognitive testing 10–20 min following the initial recall (i.e., delayed recall [40]; (2) the Word Recognition test, which assesses secondary memory by indicating if the words displayed through the Word Recall test show familiarity, representing an accuracy percentage with the number of words recognized from the original list [41]; (3) the Choice Reaction Time (CRT) test that assesses reaction time vigilance towards a target stimulus with sustained attention by scoring the accuracy of a response that correlates with its respective target [42]; (4) The Picture Recognition test, which measures secondary memory by showing a series of pictures and following the initial presentation, showing another series of images and indicating YES/NO if the image was in the initial presentation and scoring the reaction time of recognition of correct responses [43]; (5) the Digit Vigilance test, which measures the overall accuracy of correct responses to a numbering scheme presented while assessing the number of false selections as well as measuring the reaction time of correct responses [44]; (6) the Corsi Block test that evaluates present working and spatial memory by replicating a display sequence of colored blocks and is scored on the number of correct trials accurately remembered [45]; and (7) the Stroop color-word test, which measures executive function, attention, and vigilance through accuracy of overall, congruent, and incongruent stimuli by responding to a series of color names written in different colored fonts and accurately identifying the color of the font [46]. The COMPASS cognitive tests have been used in several studies and have been shown as an accurate and reliable cognitive function assessment [47,48,49].

### 3.4. Profile of Mood States

The Profile of Mood States (POMS) 65-item questionnaire evaluated changes in mood states during supplementation throughout the study. Ratings to questions were categorized into six domains (i.e., anger, confusion, depression, fatigue, tension, and vigor). Total mood disturbance score (TMDS) was determined by summing scores from anger, confusion, depression, fatigue, and tension and subtracting the vigor score. The POMS is a commonly used and valid assessment of mood states [50,51].

### 3.5. Blood Collection

A fasted blood sample was obtained from a certified phlebotomist before supplement ingestion on days 0 and 30 of the study to assess clinical health markers and safety. This involved collecting approximately 20 mL of blood from an antecubital vein into two serum separator tubes (SST) and one chelated potassium (K2) ethylenediaminetetraacetic acid (EDTA) tube (BD Vacutainer^®^, Becton, Dickinson and Company, Franklin Lakes, NJ, USA). Serum SST tubes sat for 15–30 min at room temperature and were then centrifuged (3000× *g*) at 4 °C for 10 min (MegaFuge 40R Centrifuge, Thermo Electron North America LLC, West Palm Beach, FL, USA). Serum from one SST tube was aliquoted into 1.5 mL Eppendorf storage tubes (VWR, Radnor, PA, USA) and stored at −80 °C. One SST and the EDTA tube were refrigerated until transport to Clinical Pathology Labs, Inc. (Austin, TX, USA, CLIA #45D0505003, CAP Accreditation #21525-01) for whole blood cell blood count with percent differential and serum metabolic panel analysis.

### 3.6. Side Effects Questionnaire

Perceptions of symptoms related to dietary supplementation (i.e., dizziness, headache, tachycardia, heart palpitations, dyspnea, nervousness, blurred vision, and others) were assessed using Likert-type scales as previously described [52,53].

### 3.7. Statistical Analysis

Statistical analysis software (Version 29 SPSS^®^, IBM Corp., Armonk, NY, USA) was used for statistical analysis. The sample size was selected based on our previous work [54,55,56,57] and assumed a 5% improvement with a power of 80% in primary outcome variables. Our prior worked dose effectiveness study indicated that this sample size was sufficient to determine clinically significant differences [55,56,57,58,59]. Participants were randomized into treatment groups using a balanced Latin Square designer program [60]. Repeated measures multivariate and univariate General linear model (GLM) analyses using Wilks’ Lambda and Greenhouse-Geisser statistical tests were performed on the data. Type I error was considered at a probability level of <0.05, while *p*-values between >0.05 and <0.10 are noted to identify a tendency toward significance. Pairwise comparisons were assessed using the Fisher’s least significant difference statistic. Data were also analyzed using relative dose as a covariate, but it was not significant. Mean changes from baseline with confidence intervals (CIs) of 95% assessed the clinical significance of findings. Means and CIs completely above or below baseline were considered clinically significant findings [61]. Data are shown as means and standard deviations or mean changes from baseline with lower and upper CIs (mean [LL, UL]). Partial Eta squared ($\eta_{p}^{2}$) values were used to assess effect sizes. Effect sizes were considered small (0.01), medium (0.06), and large (0.14) [62]. Ch-squared analysis was used to assess differences in categorical ratings of side effects. This statistical approach is consistent with recommendations from Earnest and colleagues [63] on best practices in reporting sport nutrition-related research.

## 4. Results

### 4.1. Demographic Data

Participant demographic data are shown in Table S1. Participants were 22.7 ± 7.4 years (range 18–49), 166.8 ± 21.7 cm, 74.9 ± 16.5 kg, and had a body mass index (BMI) of 26.2 ± 5.1 kg/m^2^. Sex differences were observed in body weight (females 68.8 ± 16.6 kg, males 82.1 ± 13.5 kg, *p* = 0.001) and resting blood pressure (females 112.3 ± 9.2 mmHg, males 125.8 ± 9.9 mmHg, *p* < 0.001). No sex differences were seen in age (*p* = 0.889), height (*p* = 0.238), BMI (*p* = 0.626), resting heart rate (*p* = 0.143), or diastolic blood pressure (*p* = 0.962).

### 4.2. Cognitive Function Assessment

#### 4.2.1. Word Recall

**Figure 3:**
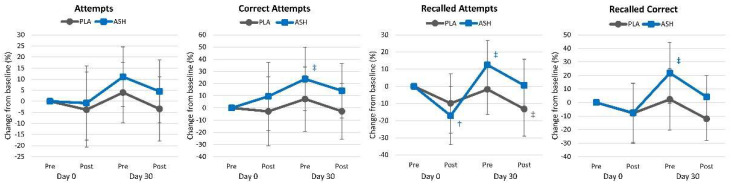
Word recall test results. † = *p* < 0.05 effect from baseline value while ‡ = *p* > 0.05 to *p* < 0.10 difference from baseline.

Table S2 presents word recall test results. Overall, multivariate Wilk’s Lambda analysis revealed a significant time effect (*p* < 0.001, ηₚ^2^ = 0.063, moderate effect) with no interaction effects observed (*p* = 0.736, ηₚ^2^ = 0.017, small effect). Univariate analysis revealed significant time effects for recall attempts (*p* = 0.010, ηₚ^2^ = 0.066, medium effect), correct attempts (*p* = 0.011, ηₚ^2^ = 0.065, medium effect), delayed recall attempts (*p* < 0.001, ηₚ^2^ = 0.155, large effect), and delayed correctly recalled (*p* < 0.001, ηₚ^2^ = 0.129, medium effect) with no significant group × time effects. Analysis of percent changes from baseline (Figure 3) revealed that after 30 days of supplementation, the number of correct attempts recalled attempts, and recalled correct attempts tended to increase above baseline with ASH supplementation, while no differences were observed in the PLA group.

#### 4.2.2. Word Recognition

**Figure 4:**
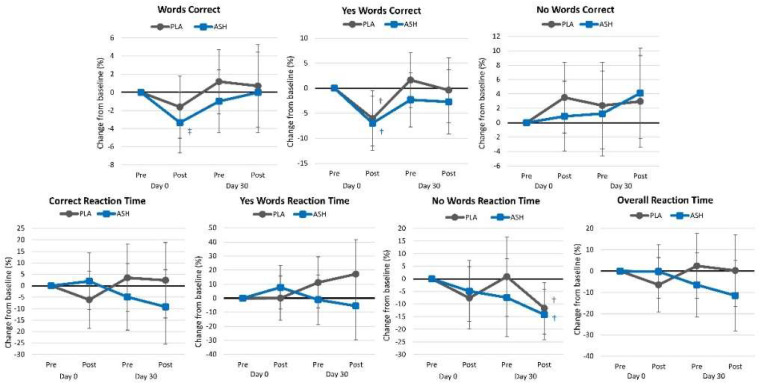
Word recognition test results. † = *p* < 0.05 difference from baseline value while ‡ = *p* > 0.05 to *p* < 0.10 difference from baseline.

Table S3 presents word recognition test results. Overall, multivariate Wilk’s Lambda analysis revealed a significant time (*p* = 0.008, ηₚ^2^ = 0.076, moderate effect) with no interaction effects observed (*p* = 0.725, ηₚ^2^ = 0.033, small effect). Univariate analysis revealed significant time effects for words correct (*p* = 0.080, ηₚ^2^ = 0.039, small effect), yes words correct (*p* = 0.003, ηₚ^2^ = 0.079, medium effect), and no words reaction time (*p* < 0.003, ηₚ^2^ = 0.104, moderate effect). No significant interaction effects were observed in words, yes, and no words correct or reaction time or overall reaction time between groups. Analysis of percent changes from baseline (Figure 4) revealed some changes from baseline values in both treatment groups after acute and 30 days of supplementation, with no significant differences observed between groups.

#### 4.2.3. Choice Reaction Time

**Figure 5:**
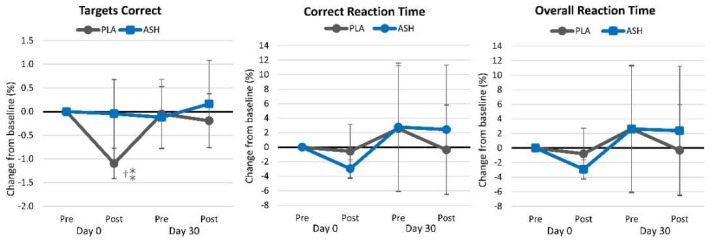
Choice reaction test results. ⁑ = *p* > 0.05 to *p* < 0.10 difference between treatment groups. † = *p* < 0.05 difference from baseline value.

Table S4 shows the choice reaction time test results. Overall, multivariate Wilk’s Lambda analysis revealed no significant time (*p* = 0.247, ηₚ^2^ = 0.022, small effect), while there tended to be an interaction effect (*p* = 0.063, ηₚ^2^ = 0.031, small effect). Univariate analysis revealed that the percent targets correct tended to change over time (*p* = 0.078, ηₚ^2^ = 0.039, small effect) while correct reaction time (*p* = 0.183, ηₚ^2^ = 0.029, small effect) and overall reaction time (*p* = 0.176, ηₚ^2^ = 0.030, small effect) were not significantly different from baseline values. Similarly, no significant interaction effects were observed in the percent targets correct (*p* = 0.118, ηₚ^2^ = 0.034, small effect), correct reaction time (*p* = 0.695, ηₚ^2^ = 0.007, small effect), or overall reaction time (*p* = 0.682, ηₚ^2^ = 0.007, small effect) on this test. Figure 5 shows the percent changes from baseline in choice reaction variables. Acute supplementation with ASH tended to maintain the ability to correctly identify targets (−0.045% [−0.81, 0.72], *p* = 0.906) while those taking the PLA observed a significant decline in the ability to correctly identify targets (−1.096% [−1.88, −0.32], *p* = 0.007) with the difference tending to differ between treatment groups (1.051% [−0.044, 2.146], *p* = 0.060). No other differences were observed over time or between treatment groups.

#### 4.2.4. Picture Recognition Test

**Figure 6:**
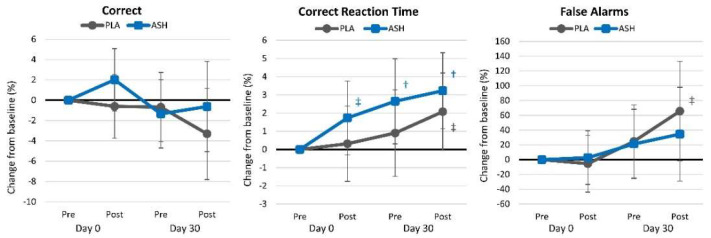
Picture recall test results. † = *p* < 0.05 difference from baseline value while ‡ = *p* > 0.05 to *p* < 0.10 difference from baseline.

Table S5 presents picture recognition test results. Overall, multivariate Wilk’s Lambda analysis revealed a significant time (*p* = 0.006, ηₚ^2^ = 0.077, moderate effect) while no significant group × time interaction effects were observed (*p* = 0.222, ηₚ^2^ = 0.049, small effect). Univariate analysis revealed time effects in the percentage of pictures correctly identified (*p* = 0.006, ηₚ^2^ = 0.086, moderate effect) while the percent of yes responses (*p* = 0.039, ηₚ^2^ = 0.052, small effect) and no response (*p* = 0.051, ηₚ^2^ = 0.054, small effect) tended to change over time. No significant group × time effects were observed in these and other measures of the picture recognition test. The pairwise comparison revealed some evidence that acute ASH ingestion improved correct reaction time (*p* = 0.006). Analysis of mean changes from baseline demonstrated that correct reaction time (*p* = 0.008) and overall reaction time (*p* = 0.023) were significantly faster in the ASH group. Figure 6 shows the percentage changes from baseline in picture recognition test variables. Results revealed that acute ASH supplementation significantly improved correct (*p* = 0.008), yes (*p* = 0.002), and overall reaction times (*p* = 0.018) and that 30 days of ASH supplementation better maintained correct picture recall, correct yes responses, correct reaction times, yes reaction time, no reaction time, and overall reaction time. Significant differences were seen between treatment groups after acute ingestion on Day 0 in correct reaction time and overall reaction time.

#### 4.2.5. Digit Vigilance

**Figure 7:**
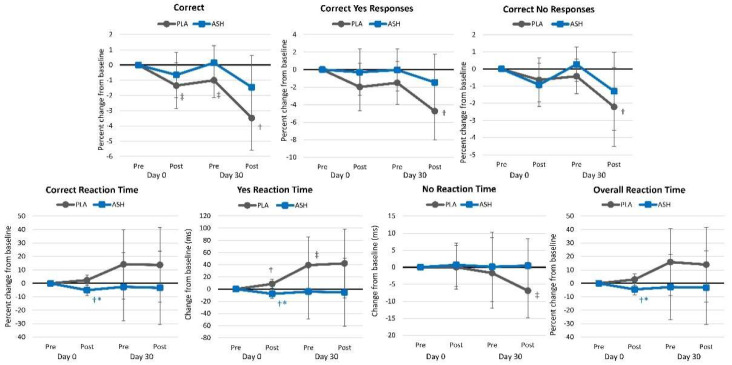
Digit vigilance test results. * = *p* < 0.05 difference between treatment groups. † = *p* < 0.05 difference from baseline value while ‡ = *p* > 0.05 to *p* < 0.10 difference from baseline.

Digit vigilance test results are presented in Table S6. Overall, multivariate Wilk’s Lambda analysis revealed a significant time (*p* = 0.006, ηₚ^2^ = 0.044, small effect) but no interaction effect (*p* = 0.698, ηₚ^2^ = 0.012, small effect). Univariate analysis revealed a significant time effect in correct response reaction time (*p* = 0.002, ηₚ^2^ = 0.087, moderate effect), while no time or group × time effects were observed in the percent targets correct or false alarm responses. Figure 7 shows the percent changes from baseline in digit vigilance test results. Correct reaction time in the ASH group increased from baseline after 30 days of supplementation (*p* = 0.042), while false alarms tended to increase over time (*p* = 0.055) in the PLA group. No significant differences were observed between treatment groups.

#### 4.2.6. Corsi Block

**Figure 8:**
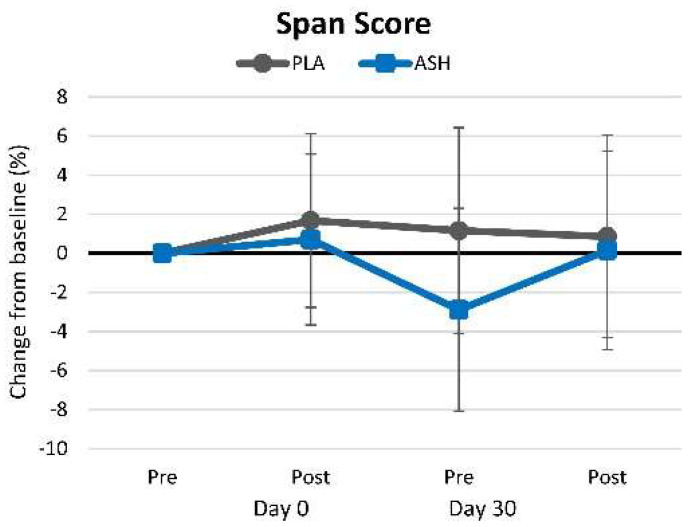
Corsi Block test results.

Table S7 shows Corsi Block test results. No significant time (*p* = 0.592, ηₚ^2^ = 0.034, small effect) or group × time (*p* = 0.754, ηₚ^2^ = 0.021, small effect) effects were observed in Corsi Block span score results. Likewise, no significant time or interaction effects were observed in the Corsi Block span score when analyzed as mean or percent changes from baseline (Figure 8).

#### 4.2.7. Stroop Test

**Figure 9:**
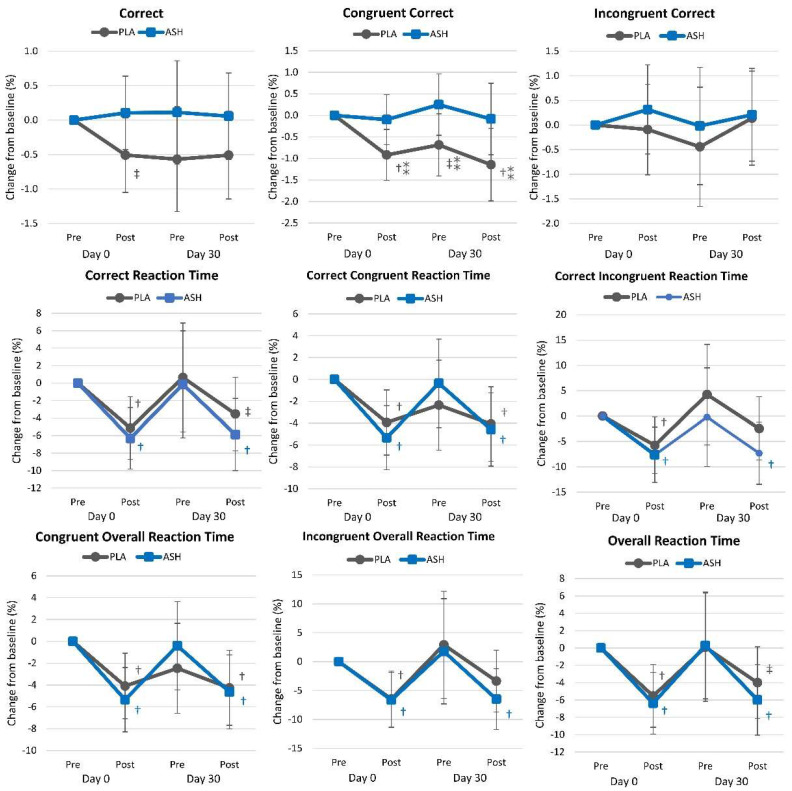
Stroop Color–Word test results. ⁑ = *p* < 0.05 difference between treatment groups. † = *p* < 0.05 difference from baseline value while ‡ = *p* > 0.05 to *p* < 0.10 difference from baseline.

Table S8 presents Stroop Color–Word test results. Overall, multivariate Wilk’s Lambda analysis revealed a significant time (*p* = 0.040, ηₚ^2^ = 0.079, moderate effect) but no interaction effect (*p* = 0.514, ηₚ^2^ = 0.051, small effect). Univariate analysis revealed significant time effects in overall reaction time (*p* < 0.001, ηₚ^2^ = 0.124, moderate effect), correct response reaction time (*p* = 0.001, ηₚ^2^ = 0.117, moderate effect), congruent words overall reaction time (*p* < 0.001, ηₚ^2^ = 0.106, moderate effect), incongruent words overall reaction time (*p* = 0.003, ηₚ^2^ = 0.099, moderate effect), and correct congruent reaction time (*p* = 0.001, ηₚ^2^ = 0.104, moderate effect) while the congruent words percent correct (*p* = 0.087, ηₚ^2^ = 0.039, small effect) and correct incongruent reaction time (*p* = 0.056, ηₚ^2^ = 0.056, small effect) tended to increase over time. However, no significant interaction effects were seen between treatments. Analysis of mean percent changes from baseline with 95% CIs (Figure 9) indicated that ASH tended to maintain the ability to identify congruent words correctly, while this ability declined in the PLA group. Reaction times improved from pre- to post-supplementation on days 0 and 30 of testing, with no differences observed between treatment groups.

### 4.3. Profile of Mood States

**Figure 10:**
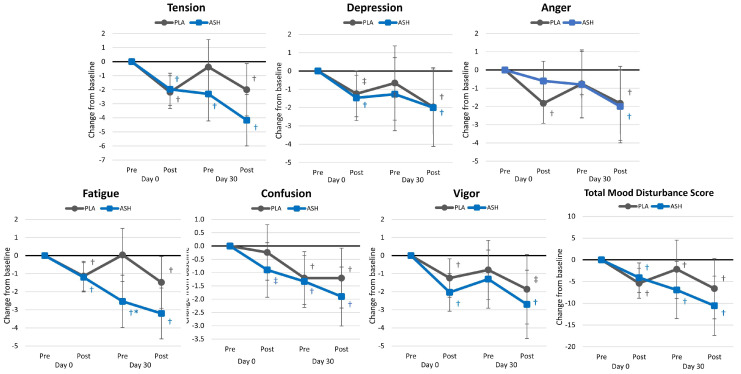
Profile of Mood States results. * = *p* > 0.05 to *p* < 0.10 difference between treatment groups. † = *p* < 0.05 difference from baseline value while ‡ = *p* > 0.05 to *p* < 0.10 difference from baseline.

Table S9 shows the results of the POMS questionnaire. Overall, multivariate Wilk’s Lambda analysis indicated a time (*p* < 0.001, ηₚ^2^ = 0.148, large effect) but no interaction effects observed (*p* = 0.148, ηₚ^2^ = 0.047, small effect). Univariate analysis revealed significant time effects in tension (*p* < 0.001, ηₚ^2^ = 0.189, large effect), depression (*p* < 0.001, ηₚ^2^ = 0.120, large effect), anger (*p* < 0.001, ηₚ^2^ = 0.139, large effect), fatigue (*p* < 0.001, ηₚ^2^ = 0.200, large effect), confusion (*p* < 0.001, ηₚ^2^ = 0.145 large effect), vigor (*p* = 0.004, ηₚ^2^ = 0.098, moderate effect, and TMDS (*p* < 0.001, ηₚ^2^ = 0.188, large effect). Fatigue levels tended to interact (*p* = 0.058, ηₚ^2^ = 0.047, small effect) with no other group × time effects observed. Analysis of changes from baseline (Figure 10) revealed evidence that participants in the ASH experienced a decrease in tension scores from baseline after acute (*p* = 0.001), 30 days (*p* = 0.01), and acute ingestion after 30 days (*p* < 0.001) of supplementation while tension scores were not significantly different from baseline values after 30 days of supplementation. Additionally, there was evidence that supplementation with ASH for 30 days reduced perceptions of fatigue after acute supplementation on Day 0 (*p* = 0.005) and Day 30 (*p* = 0.002) with 30 Day Pre values significantly lower in the ASH group compared to the PLA group (−1.91 [−5.6, −0.3], *p* = 0.023). No other significant differences were observed between treatment groups in the remaining POMS variables.

### 4.4. Markers of Health and Safety

Overall GLM analysis of cell blood count data (Table S10) revealed no significant time (*p* = 0.120, ηₚ^2^ = 0.336, large effect) or group × time effect (*p* = 0.341, ηₚ^2^ = 0.269, large effect). Univariate analysis revealed a significant interaction effect in hematocrit (*p* = 0.004), where hematocrit decreased in the PLA group while maintained in the ASH group. The pairwise comparison also revealed no differences between treatment groups in baseline cell blood counts. However, lymphocytes in the ASH group were significantly lower than in the PLA group after 30 days of ASH supplementation. With ASH supplementation, red blood cells, neutrophils, and platelets tended to be higher. Overall GLM analysis of standard serum clinical markers (Table S11) revealed a trend toward a time effect (*p* = 0.0065, ηₚ^2^ = 0.554, large effect) with no interaction effects (*p* = 0.150, ηₚ^2^ = 0.509, large effect). Univariate analysis revealed significant time effects in several variables, with interaction effects observed in high-density lipoprotein (*p* = 0.011), protein (*p* = 0.005), albumin (*p* = 0.027), alkaline phosphatase (*p* = 0.002), and estimated glomerular filtration rate (*p* = 0.037). Participants in the ASH group observed a significant increase in high-density lipoprotein (HDL) cholesterol and a decrease in the HDL to total cholesterol ratio from baseline, while the blood urea nitrogen (BUN) to creatinine ratio increased significantly from baseline in the PLA group. There was also evidence that BUN and total bilirubin levels were lower and total protein and calcium levels higher after supplementation with ASH. Collectively, although differences over time and between treatment groups were small and within normal clinical values, results suggest that clinical blood profiles more favorably improved with ASH supplementation. Finally, Table S12 presents the side effects evaluated during the study. No significant differences in the frequency or severity of side effects evaluated or any other complaints. No participant withdrew from the study due to any perceived side effects.

## 5. Discussion

Ashwagandha has been reported to possess antioxidant [9,14,15,16], anti-inflammatory [9,11,12], neuroprotective [8,10,15,23,24,25,26], endocrinological [17,18,19,20,21], and immune-modulatory properties [10,22] that influence cognition [1,23,25,31]. Preliminary human clinical trials indicate that ashwagandha supplementation may have therapeutic benefits in individuals with medically managed bipolar disease [32], mild cognitive impairment [33], and chronic stress [7]. There is also evidence that ashwagandha supplementation improves memory, reaction times, and psychomotor performance in healthy participants [35,37,38]. Additionally, there is evidence that 30 days of ashwagandha supplementation improved perceptions of energy, sleep quality, and well-being in college students [36]. However, more research is needed to assess the potential nootropic effects of ashwagandha on cognitive function. Results from this study provide additional evidence that acute and 30 days of liposomal ashwagandha supplementation affect cognitive function and mood in healthy men and women. The following provides additional insight regarding the observed results.

### 5.1. Primary Outcomes

We evaluated the effects of ashwagandha supplementation on several cognitive function assessments to assess episodic memory (word presentation, picture recognition, delayed word recall, delayed picture recognition, delayed word recognition), attention and vigilance (simple reaction times, digit vigilance test, choice reaction time), and executive function (Stroop color-word test) [4]. Consistent with our prior work [37], we found evidence that acute ingestion of liposomal ashwagandha affected cognitive function in healthy individuals at a lower dose as previously established (225 mg compared to 400 mg). In this regard, present findings indicated that acute ashwagandha supplementation maintained the ability to identify correct targets on the choice reaction test, improved picture recognition reaction time, and prevented a decline in correct responses on the Stroop test. After 30 days of supplementation, there was some evidence that word recall correct attempts, correct reaction time in the digit vigilance task test, and Stroop test congruent correct responses improved to a greater degree from baseline with ashwagandha supplementation. Moreover, POMS tension and fatigue scores decreased from baseline, with Pre 30 Day fatigue scores in the ashwagandha group significantly lower than placebo responses. Collectively, these findings provide additional evidence that acute and longer periods of ashwagandha supplementation can improve measures of episodic memory, attention and vigilance, and executive function, as well as perceptions of tension and fatigue. These findings support reports that ashwagandha supplementation improved reaction times and cognitive performance in healthy young males [35], perceptions of energy and mental clarity in college students [36], and that 30 days of liposomal ashwagandha supplementation (225 or 400 mg/d) reduced stress-related markers and improved cognitive function in individuals with perceived stress [34].

While not directly assessed in the present study, there are several potential mechanisms described in the literature in which ashwagandha may influence cognition. First, ashwagandha has been reported to serve as an adaptogen, thereby improving response and resilience to stress [2,3,11,14,36,64,65]. The cognitive function tests produce a stressful state. Consequently, ashwagandha may have improved cognitive function by allowing the participants to perform better during stressful situations. Second, ashwagandha has been reported to inhibit acetylcholinesterase, increase neurotransmission [11,14,65], and provide neuroprotective effects [8,15,25,65,66]. Ashwagandha has also been reported to influence gamma-aminobutyric acid (GABA) activity [1,67,68,69]. GABA is an inhibitory neurotransmitter that has been a target in the treatment of anxiety [69]. Ashwagandha has been reported to have GABAergic activity and have similar effects as anxiolytic pharmaceutical interventions [69]. It has also been reported to promote the quality of sleep [68]. Thus, ashwagandha supplementation may help individuals in stressful conditions manage anxiety and/or improve the quality of sleep, thereby improving cognitive function. Finally, ashwagandha has also been reported to reduce oxidative stress [9,16] and inflammation [8,15,22,25]. Oxidative stress and inflammation in the brain have been implicated in the progression of cognitive impairment during aging. While we studied younger participants, it is possible that ashwagandha mediated oxidative stress and/or inflammation, thereby improving cognitive function. Additional research should examine the role of ashwagandha supplementation on these mechanisms, memory, and cognitive function in younger and older populations under stressful conditions who might benefit.

### 5.2. Secondary Outcomes

Since ashwagandha has antioxidant [9,14,15,16], endocrine [17,18,19,20,21], immune [10,22], and anti-inflammatory effects [9,11,12], there has been interest in determining the effects of ashwagandha supplementation on markers of health and safety. In this study, we assessed the effects of 30 days of ashwagandha supplementation on a comprehensive panel of whole blood and serum health markers and perceptions about the frequency and severity of common side effects. Results revealed that ashwagandha supplementation either had no effect or more favorably improved clinical blood panels with no differences in reported perceptions of side effects. These findings are consistent with results from Chandrasekhar et al. [6], who reported that ashwagandha supplementation (2 × 300 mg/d for 60 days) in individuals with a history of stress and anxiety had no clinically significant effects on clinical blood parameters or reported side effects. Additionally, results support findings from Smith and coworkers [18], who reported that 2 × 200 mg/d of ashwagandha root extract supplementation for 12 weeks in overweight men and women between the ages of 40–75 years experiencing high levels of fatigue and stress did not significantly affect blood glucose, HbA1c, cell blood counts, markers liver function, markers of renal function, or perceptions of side effects. Current findings also support other studies reporting that ashwagandha supplementation (500–700 mg/d for 2–8 weeks) in adults was well-tolerated and did not result in perceptions of side effects [33,64,70,71].

### 5.3. Limitations and Future Directions

Although positive results were observed, there are several limitations that should be considered. First, although participants maintained a similar diet, activity level, and fasting state before each testing session, diet and exercise were not controlled during the entire study. It is possible that variations in physical activity may have affected fatigue perception and/or ingestion of various stimulants in the participant’s normal diet may have affected results. Second, we performed a single-dose effectiveness study and established that ingestion of 400 mg of liposomal ashwagandha improved some measures of memory, attention, and reaction time for several hours after ingestion [37]. Additionally, Remenapp et al. [34] reported that dietary supplementation of 225 mg/d with this source of liposomal ashwagandha improved cognitive function measures in individuals with perceived stress. Present findings indicate that younger healthy individuals may also benefit from supplementing their diet with 225 mg/d of liposomal ashwagandha. However, it is possible that ingesting higher amounts of ashwagandha, several doses a day as has been previously investigated, or a longer period of supplementation may have promoted greater effects. Consequently, additional research is warranted to (1) conduct studies on more frequent, higher doses, and/or a longer supplementation period; (2) evaluate the effects of ashwagandha supplementation during a standardized and supervised exercise and diet intervention; (3) examine the potential nootropic effects of ashwagandha supplementation in older individuals as a nutrition strategy to maintain cognitive function before experiencing clinically significant mild cognitive impairment, (4) assessing the effects of ashwagandha supplementation on managing stress and improving quality of life as people age; and, the potential role of ashwagandha supplementation in improving memory, attention, and executive function in individuals with signs of memory and cognitive impairment.

## 6. Conclusions

This study’s results support that acute (225 mg) and 30 days of dietary supplementation of a liposomal form of ashwagandha (225 mg/d) can improve short-term memory, attention and vigilance, and reaction times in healthy younger men and women. While these results are promising, additional research is warranted to evaluate the potential nootropic effects of ashwagandha at different dosing strategies, longer supplementation periods, older individuals to help maintain cognitive function as people age, and potential clinical applications in individuals experiencing stressful conditions, memory and/or cognitive decline.

## Data Availability

Data and statistical analyses are available upon request on a case-by-case basis for non-commercial scientific inquiry and educational use if IRB restrictions and research agreement terms are not violated.

## Supplementary Materials

The following supporting information can be downloaded at: https://www.mdpi.com/article/10.3390/nu16121813/s1, Table S1: Participant Demographic Data, Table S2: Word Recall test results, Table S3: Word Recognition test results, Table S4: Choice Reaction Time (CRT) test results, Table S5: Picture Recognition test results, Table S6: Digit Vigilance test results, Table S7: Corsi Block test results, Table S8: Stroop Color Word test results, Table S9: Profile of Mood States results, Table S10: Complete Blood Count data, Table S11: Serum Metabolic Panel, Table S12: Frequency and Severity of Side Effects.
