# Supplementary material for: Acute and Repeated Ashwagandha Supplementation Improves Markers of Cognitive Function and Mood
Source: Nutrients. 2024 Jun 8;16(12):1813. doi: 10.3390/nu16121813 (PMC11207027; doi:10.3390/nu16121813)
Supplement: Supplementary file 1 [file nutrients-16-01813-s001.zip › nutrients-3036936-supplementary.pdf]

Table S1: Participant Demographic Data.

Variable	Group	*N*	Female	Male	Total	Mean (SEM)	Effect	p-Level	η_p^2
Age (yrs)	PL	*29*	21.3 ± 3.1	23.8 ± 10.7	22.4 ± 7.5	22.5 ± 1.4	Group	0.845	0.001
	ASH	*30*	24.5 ± 9.9	21.4 ± 1.7	23.0 ± 7.4	22.9 ± 1.4	Sex	0.889	0.000
	Total	*59*	22.9 ± 7.4	22.6 ± 7.5	22.7 ± 7.4	22.7 ± 1.0	G x S	0.145	0.038
Height (cm)	PL	*29*	161.7 ± 6.2	162.5 ± 43.9	162.0 ± 29.1	162.1 ± 4.0	Group	0.087	0.052
	ASH	*30*	165.6 ± 6.6	178.0 ± 6.9	171.4 ± 9.1	171.8 ± 3.9	Sex	0.238	0.025
	Total	*59*	163.6 ± 6.6	170.5 ± 31.2 ⁑	166.8 ± 21.7	166.9 ± 2.8 ⁑	G x S	0.300	0.020
Weight (kg)	PL	*29*	62.2 ± 11.7	83.3 ± 15.4	71.7 ± 17.0	72.8 ± 2.7 ◊	Group	0.166	0.035
	ASH	*30*	75.3 ± 18.3	81.0 ± 11.9	78.0 ± 15.7	78.1 ± 2.7	Sex	0.001	0.182
	Total	*59*	68.8 ± 16.6 ∗	82.1 ± 13.5	74.9 ± 16.5	75.5 ± 1.9 ◊	G x S	0.048	0.069
Body Mass Index (kg/m2)	PL	*29*	23.9 ± 4.7	27.4 ± 4.9	25.5 ± 5.1	25.7 ± 0.9 ♦	Group	0.427	0.012
	ASH	*30*	27.8 ± 6.3	25.6 ± 3.3	26.8 ± 5.2	26.7 ± 0.9	Sex	0.626	0.004
	Total	*59*	25.9 ± 5.9 ∗	26.5 ± 4.2	26.2 ± 5.1	26.2 ± 0.7	G x S	0.030	0.082
Resting Heart Rate (bpm)	PL	*29*	68.8 ± 10.8	67.8 ± 8.0	68.4 ± 9.5	68.3 ± 2.1	Group	0.902	0.000
	ASH	*30*	72.7 ± 15.1	64.7 ± 10.1	69.0 ± 13.4	68.7 ± 2.1 ♦	Sex	0.143	0.039
	Total	*59*	70.8 ± 13.0	66.2 ± 9.2	68.7 ± 11.6	68.5 ± 1.5	G x S	0.249	0.024
Resting Systolic Blood Pressure (mmHg)	PL	*29*	108.3 ± 7.7	126.4 ± 11.8	116.4 ± 13.2	117.3 ± 1.7 ◊	Group	0.161	0.035
	ASH	*30*	116.3 ± 9.0	125.2 ± 8.2	120.5 ± 9.6	120.8 ± 1.7 ◊	Sex	0.000	0.364
	Total	*59*	112.3 ± 9.2 ∗	125.8 ± 9.9	118.5 ± 11.6	119.1 ± 1.2 ◊	G x S	0.062	0.062
Resting Diastolic Blood Pressure (mmHg)	PL	*29*	70.4 ± 5.5	71.4 ± 5.7	70.9 ± 5.5	70.9 ± 1.3	Group	0.010	0.115
	ASH	*30*	76.1 ± 9.4	75.3 ± 5.5	75.7 ± 7.7	75.7 ± 1.3	Sex	0.962	0.000
	Total	*59*	73.3 ± 8.1 ∗	73.4 ± 5.8	73.3 ± 7.1	73.3 ± 0.9 ∗	G x S	0.631	0.004

Data are expressed as means ± standard deviations for the placebo (PL) and Ashwagandha (ASH) groups. Data were analyzed using a multivariate and univariate General Linear Model with repeated measures. P-levels, with partial ETA squared (η_p^2), are listed for between-subject group (G), sex (S), and group x sex (G x S) effects. Multivariate Wilk's Lambda showed significant group ($p = 0.028$, $\eta_p^2 = 0.264$), sex ($p<0.001$, $\eta_p^2 = 0.638$), but not group x sex ($p = 0.068$, $\eta_p^2 = 0.226$) within-subject effects. Pairwise comparisons, with LSD confidence interval adjustment, for simple main effects are indicated by the following superscripts: difference between groups ∗ = p<0.05 (⁑ = p>0.05 to p<0.10); and between sexes, ◊ = p<0.05 (♦ = p>0.05 to p<0.10). η_p^2 effect size values of 0.01 - 0.05 = small, 0.06 - 0.13 = medium, and >0.14 = large.

Table S2: Word Recall test results.

Variable	Group	N	Day 0 Pre	Day 0 Post	Day 30 Pre	Day 30 Post	Mean (SEM)	Effect	p-Level	η_p^2
Recall Attempts	PL	*29*	7.48 ± 1.50	7.07 ± 1.89	7.66 ± 1.61	7.17 ± 2.07	7.35 ± 0.29	Group	0.842	0.001
	ASH	*30*	7.80 ± 2.57	6.73 ± 2.07 †	7.87 ± 2.03	7.30 ± 2.14	7.43 ± 0.28	Time	0.010	0.066
	Time	*59*	7.64 ± 2.10	6.90 ± 1.97 †	7.76 ± 1.82	7.24 ± 2.09	7.39 ± 0.20	G x T	0.636	0.009
Correct Attempts	PL	*29*	7.14 ± 1.68	6.69 ± 1.89	7.38 ± 1.72	6.79 ± 2.21	7.00 ± 0.31	Group	0.834	0.001
	ASH	*30*	7.13 ± 2.76	6.13 ± 2.24 †	7.40 ± 2.22	6.97 ± 2.46	6.91 ± 0.31	Time	0.011	0.065
	Time	*59*	7.14 ± 2.28	6.41 ± 2.08 †	7.39 ± 1.97	6.88 ± 2.32	6.95 ± 0.22	G x T	0.616	0.010
Delayed Recall Attempts	PL	*29*	6.79 ± 2.11	5.62 ± 2.03 †	6.38 ± 1.70	5.66 ± 2.21 †	6.11 ± 0.31	Group	0.827	0.001
	ASH	*30*	6.47 ± 2.39	5.00 ± 1.93 †	6.63 ± 2.24	5.97 ± 2.08	6.02 ± 0.30	Time	0.000	0.155
	Time	*59*	6.63 ± 2.24	5.31 ± 1.98 †	6.51 ± 1.98	5.81 ± 2.13 †	6.06 ± 0.22	G x T	0.245	0.024
Delayed Correctly Recalled	PL	*29*	6.24 ± 1.98	5.24 ± 2.13 †	6.07 ± 1.73	5.21 ± 2.09 †	5.69 ± 0.33	Group	0.555	0.006
	ASH	*30*	5.70 ± 2.58	4.47 ± 2.01 †	6.07 ± 2.55	5.43 ± 2.36	5.42 ± 0.32	Time	0.000	0.129
	Time	*59*	5.97 ± 2.30	4.85 ± 2.09 †	6.07 ± 2.16	5.32 ± 2.22 †	5.55 ± 0.23	G x T	0.250	0.024

Data are expressed as means ± standard deviations for the placebo (PL) and Ashwagandha (ASH) groups. Data were analyzed using a multivariate and univariate General Linear Model with repeated measures. P-levels, with partial ETA squared (η_p^2), are listed for between-subject group (G) and univariate within-subject (Greenhouse-Geisser) time (T), and group x time (G x T) effects. Multivariate Wilk's Lambda showed significant time ($p<0.001$, $\eta_p^2 = 0.063$), but no group x time ($p = 0.736$, $\eta_p^2 = 0.017$), within-subject effects. Pairwise comparisons, with LSD confidence interval adjustment, for simple main effects are indicated by the following superscripts: difference from baseline value, † = p<0.05 (‡ = p>0.05 to p<0.10); and between groups ∗ = p<0.05 (⁑ = p>0.05 to p<0.10). η_p^2 effect size values of 0.01 - 0.05 = small, 0.06 - 0.13 = medium, and >0.14 = large.

Table S3: Word Recognition test results.

Variable	Group	N	Day 0 Pre	Day 0 Post	Day 30 Pre	Day 30 Post	Mean (SEM)	Effect	p-Level	η^2
Words Correct (%)	PL	*29*	91.03 ± 7.19	89.20 ± 7.75	91.72 ± 6.28	91.15 ± 8.37	90.78 ± 1.01	Group	0.353	0.015
	ASH	*30*	90.89 ± 8.75	87.44 ± 7.96 †	89.33 ± 6.91	90.11 ± 7.08	89.44 ± 1.00	Time	0.080	0.039
	Time	*59*	90.96 ± 7.95	88.31 ± 7.84 †	90.51 ± 6.66	90.62 ± 7.69	90.11 ± 0.71	G x T	0.758	0.007
Overall Reaction Time (milliseconds)	PL	*29*	1053 ± 258	957 ± 227	1048 ± 531	1024 ± 640	1021 ± 73	Group	0.803	0.001
	ASH	*30*	1169 ± 695	1083 ± 567	1004 ± 425 ‡	929 ± 275 †	1046 ± 72	Time	0.176	0.030
	Time	*59*	1112 ± 527	1021 ± 436	1026 ± 476	975 ± 488 ‡	1033 ± 51	G x T	0.183	0.029
Correct Reaction Time (milliseconds)	PL	*29*	998 ± 242	915 ± 228	1012 ± 511	998 ± 643	981 ± 73	Group	0.757	0.002
	ASH	*30*	1115 ± 705	1055 ± 569	973 ± 431	908 ± 264 ‡	1013 ± 72	Time	0.350	0.018
	Time	*59*	1057 ± 530	986 ± 438	992 ± 469	952 ± 486	997 ± 51	G x T	0.168	0.031
YES Words Correct (%)	PL	*29*	87.59 ± 9.38	82.07 ± 15.13 †	88.51 ± 10.97	86.90 ± 13.74	86.26 ± 1.66	Group	0.556	0.006
	ASH	*30*	88.44 ± 11.99	81.56 ± 12.46 †	85.11 ± 10.46	84.44 ± 11.26	84.89 ± 1.63	Time	0.003	0.079
	Time	*59*	88.02 ± 10.71	81.81 ± 13.72 †	86.78 ± 10.76	85.65 ± 12.49	85.58 ± 1.16	G x T	0.597	0.011
NO Words Correct (%)	PL	*29*	94.48 ± 10.70	96.32 ± 5.22	94.94 ± 7.70	95.40 ± 6.20	95.29 ± 1.04	Group	0.382	0.013
	ASH	*30*	93.33 ± 11.21	93.33 ± 8.40	93.55 ± 8.84	95.78 ± 5.93	94.00 ± 1.02	Time	0.562	0.011
	Time	*59*	93.90 ± 10.88	94.80 ± 7.12	94.24 ± 8.26	95.59 ± 6.01	94.64 ± 0.73	G x T	0.611	0.010
YES Words Reaction Time (milliseconds)	PL	*29*	1002 ± 285	967 ± 301	1073 ± 668	1137 ± 1079	1045 ± 90	Group	0.856	0.001
	ASH	*30*	1144 ± 692	1164 ± 783	999 ± 420	963 ± 320	1068 ± 88	Time	0.927	0.001
	Time	*59*	1074 ± 533	1067 ± 600	1036 ± 552	1049 ± 788	1056 ± 63	G x T	0.125	0.036
NO Words Reaction Time (milliseconds)	PL	*29*	1105 ± 436	955 ± 191	1023 ± 436	910 ± 236 ‡	998 ± 64	Group	0.766	0.002
	ASH	*30*	1194 ± 762	1002 ± 418 †	1010 ± 468 ‡	894 ± 258 †	1025 ± 63	Time	0.003	0.104
	Time	*59*	1150 ± 620	979 ± 325 †	1017 ± 449 ‡	902 ± 246 †	1012 ± 45	G x T	0.655	0.007

Data are expressed as means ± standard deviations for the placebo (PL) and Ashwagandha (ASH) groups. Data were analyzed using a multivariate and univariate General Linear Model with repeated measures. P-levels, with partial ETA squared (η^2), are listed for between-subject group (G) and univariate within-subject (Greenhouse-Geisser) time (T), and group x time (G x T) effects. Multivariate Wilk's Lambda showed significant time ($p = 0.008$, $\eta^2 = 0.076$), but no group x time ($p = 0.725$, $\eta^2 = 0.033$), within-subject effects. Pairwise comparisons, with LSD confidence interval adjustment, for simple main effects are indicated by the following superscripts: difference from baseline value, † = p<0.05 (‡ = p>0.05 to p<0.10); and between groups ∗ = p<0.05 (⁑ = p>0.05 to p<0.10). η^2 effect size values of 0.01 - 0.05 = small, 0.06 - 0.13 = medium, and >0.14 = large.

Table S4: Choice Reaction Time (CRT) test results.

			Day 0			Day 30		Mean			
**Variable**	**Group**	**N**	**Pre**	**Post**		**Pre**	**Post**	**(SEM)**	**Effect**	**p-Level**	**η^2**
Targets Correct	PL	*29*	98.83 ± 1.47	97.72 ± 1.75	†	98.76 ± 1.64	98.62 ± 1.78	98.48 ± 0.24	Group	0.921	0.000
(%)	ASH	*30*	98.47 ± 1.87	98.40 ± 1.61		98.33 ± 2.17	98.60 ± 1.50	98.45 ± 0.23	Time	0.078	0.039
	Time	*59*	98.64 ± 1.68	98.07 ± 1.70	†	98.54 ± 1.92	98.61 ± 1.63	98.47 ± 0.17	G x T	0.118	0.034
Overall Reaction Time	PL	*29*	496.4 ± 106.1	485.6 ± 73.9		517.3 ± 219.1	491.1 ± 106.0	497.6 ± 17.5	Group	0.864	0.001
(milliseconds)	ASH	*30*	494.0 ± 71.8	474.7 ± 56.9		502.7 ± 81.4	501.9 ± 106.0	493.4 ± 17.2	Time	0.176	0.030
	Time	*59*	495.2 ± 89.5	480.1 ± 65.5	‡	509.9 ± 162.9	496.6 ± 105.2	495.5 ± 12.3	G x T	0.695	0.007
Correct Reaction Time	PL	*29*	497.7 ± 106.7	487.9 ± 74.5		518.1 ± 218.8	491.8 ± 105.4	498.9 ± 17.5	Group	0.863	0.001
(milliseconds)	ASH	*30*	495.1 ± 71.4	475.6 ± 57.2		504.5 ± 81.7	503.2 ± 105.9	494.6 ± 17.2	Time	0.183	0.029
	Time	*59*	496.3 ± 89.7	481.6 ± 66.0	‡	511.2 ± 162.8	497.6 ± 104.9	496.7 ± 12.3	G x T	0.682	0.007

Data are expressed as means ± standard deviations for the placebo (PL) and Ashwagandha (ASH) groups. Data were analyzed using a multivariate and univariate General Linear Model with repeated measures. P-levels, with partial ETA squared (η^2), are listed for between-subject group (G) and univariate within-subject (Greenhouse-Geisser) time (T), and group x time (G x T) effects. Multivariate Wilk's Lambda showed no significant time ($p = 0.247$, $\eta^2 = 0.022$), or group x time ($p = 0.063$, $\eta^2 = 0.031$), within-subject effects. Pairwise comparisons, with LSD confidence interval adjustment, for simple main effects are indicated by the following superscripts: difference from baseline value, † = $p<0.05$ (‡ = $p>0.05$ to $p<0.10$); and between groups ∗ = $p<0.05$ (⁑ = $p>0.05$ to $p<0.10$). η^2 effect size values of 0.01 - 0.05 = small, 0.06 - 0.13 = medium, and >0.14 = large.

Table S5: Picture Recognition test results.

Variable	Group	N	Day 0 Pre	Day 0 Post	Day 30 Pre	Day 30 Post	Mean (SEM)	Effect	p-Level	η_p^2
Pictures Correct (%)	PL	*29*	98.74 ± 2.26	97.36 ± 3.92 ‡	97.70 ± 2.69 ‡	95.29 ± 7.21 †	97.27 ± 0.42	Group	0.184	0.031
	ASH	*30*	98.56 ± 2.09	97.89 ± 3.44	98.67 ± 2.25	97.11 ± 3.79	98.06 ± 0.41	Time	0.006	0.086
	Time	*59*	98.64 ± 2.15	97.63 ± 3.66 ‡	98.19 ± 2.50	96.21 ± 5.76 †	97.66 ± 0.29	G x T	0.436	0.014
Overall Reaction Time (milliseconds)	PL	*29*	814 ± 88	833 ± 120	960 ± 891	944 ± 993	888 ± 66	Group	0.368	0.014
	ASH	*30*	829 ± 135	790 ± 144 †	801 ± 127	795 ± 111	804 ± 65	Time	0.493	0.009
	Time	*59*	822 ± 113	811 ± 133	879 ± 631	868 ± 698	846 ± 46	G x T	0.396	0.013
Correct Reaction Time (milliseconds)	PL	*29*	804 ± 76	822 ± 116	938 ± 924	936 ± 995	875 ± 67	Group	0.416	0.012
	ASH	*30*	826 ± 134	778 ± 105 †	800 ± 129	791 ± 108	799 ± 65	Time	0.497	0.008
	Time	*59*	815 ± 109	800 ± 112	868 ± 652	862 ± 699	837 ± 47	G x T	0.428	0.011
Yes Targets Correct (%)	PL	*29*	98.16 ± 3.94	96.09 ± 7.24	96.55 ± 5.53	93.56 ± 10.91 †	96.09 ± 0.74	Group	0.196	0.029
	ASH	*30*	98.00 ± 3.97	97.56 ± 5.10	97.78 ± 4.41	96.44 ± 6.25	97.44 ± 0.73	Time	0.039	0.052
	Time	*59*	98.08 ± 3.92	96.84 ± 6.23	97.17 ± 4.98	95.03 ± 8.89 †	96.77 ± 0.52	G x T	0.508	0.013
No Targets Correct (%)	PL	*29*	99.31 ± 2.73	98.62 ± 3.28	98.85 ± 2.56	97.01 ± 6.57 †	98.45 ± 0.39	Group	0.769	0.002
	ASH	*30*	99.11 ± 2.31	98.22 ± 4.27	99.33 ± 2.04	97.78 ± 4.04	98.61 ± 0.39	Time	0.051	0.054
	Time	*59*	99.21 ± 2.50	98.42 ± 3.78 ‡	99.10 ± 2.30	97.40 ± 5.40 †	98.53 ± 0.28	G x T	0.667	0.006
Yes Targets Reaction Time (milliseconds)	PL	*29*	735 ± 100	789 ± 156 ‡	1038 ± 1407	1064 ± 1741	907 ± 104	Group	0.389	0.013
	ASH	*30*	829 ± 176	746 ± 111 †	779 ± 147	766 ± 121	780 ± 102	Time	0.356	0.015
	Time	*59*	783 ± 150 ∗	767 ± 136	906 ± 992	913 ± 1222	843 ± 73	G x T	0.280	0.021
N0 Targets Reaction Time (milliseconds)	PL	*29*	892 ± 143	877 ± 151	881 ± 396	823 ± 268 ‡	869 ± 33	Group	0.386	0.013
	ASH	*30*	830 ± 153	834 ± 214	824 ± 182	824 ± 160	828 ± 33	Time	0.519	0.012
	Time	*59*	860 ± 150	855 ± 185	852 ± 305	824 ± 218	848 ± 23	G x T	0.610	0.009

Data are expressed as means ± standard deviations for the placebo (PL) and Ashwagandha (ASH) groups. Data were analyzed using a multivariate and univariate General Linear Model with repeated measures. P-levels, with partial ETA squared (η_p^2), are listed for between-subject group (G) and univariate within-subject (Greenhouse-Geisser) time (T), and group x time (G x T) effects. Multivariate Wilk's Lambda showed significant time ($p = 0.006$, $\eta_p^2 = 0.077$), but no group x time ($p = 0.222$, $\eta_p^2 = 0.049$), within-subject effects. Pairwise comparisons, with LSD confidence interval adjustment, for simple main effects are indicated by the following superscripts: difference from baseline value, † = $p<0.05$ (‡ = $p>0.05$ to $p<0.10$); and between groups ∗ = $p<0.05$ (⁑ = $p>0.05$ to $p<0.10$). η_p^2 effect size values of 0.01 - 0.05 = small, 0.06 - 0.13 = medium, and >0.14 = large.

Table S6: Digit Vigilance test results.

Variable	Group	N	Day 0 Pre	Day 0 Post	Day 30 Pre	Day 30 Post	Mean (SEM)	Effect	p-Level	η^2
Targets Correct	PL	*29*	90.19 ± 6.71	89.35 ± 5.52	89.27 ± 7.10	86.82 ± 11.22 ‡	88.91 ± 1.39	Group	0.106	0.045
(%)	ASH	*30*	85.93 ± 9.84	87.19 ± 9.05	84.52 ± 10.71	85.19 ± 11.99	85.70 ± 1.37	Time	0.194	0.028
	Time	*59*	88.02 ± 8.65 ⁑	88.25 ± 7.54	86.86 ± 9.35 ⁑	85.99 ± 11.55	87.31 ± 0.98	G x T	0.443	0.015
Correct Reaction Time	PL	*29*	486.0 ± 34.2	486.7 ± 29.4	490.2 ± 36.2	495.7 ± 35.4 †	489.6 ± 5.8	Group	0.633	0.004
(ms)	ASH	*30*	485.9 ± 43.6	492.3 ± 29.3	496.4 ± 32.6 †	499.5 ± 30.8 †	493.5 ± 5.7	Time	0.002	0.087
	Time	*59*	486.0 ± 38.9	489.5 ± 29.2	493.3 ± 34.3 †	497.6 ± 32.9 †	491.6 ± 4.1	G x T	0.700	0.008
False Alarms	PL	*29*	2.62 ± 2.35	2.28 ± 1.89	2.62 ± 2.27	3.31 ± 2.87	2.71 ± 0.39	Group	0.139	0.038
	ASH	*30*	3.80 ± 3.20	3.07 ± 2.96 ‡	3.47 ± 2.66	3.80 ± 3.21	3.53 ± 0.39	Time	0.116	0.035
	Time	*59*	3.22 ± 2.85	2.68 ± 2.50 ‡	3.05 ± 2.49	3.56 ± 3.03	3.12 ± 0.28	G x T	0.805	0.005

Data are expressed as means ± standard deviations for the placebo (PL) and Ashwagandha (ASH) groups. Data were analyzed using a multivariate and univariate General Linear Model with repeated measures. P-levels, with partial ETA squared (η^2), are listed for between-subject group (G) and univariate within-subject (Greenhouse-Geisser) time (T), and group x time (G x T) effects. Multivariate Wilk's Lambda showed significant time ($p = 0.006$, $\eta^2 = 0.044$), but not group x time ($p = 0.698$, $\eta^2 = 0.012$), within-subject effects. Pairwise comparisons, with LSD confidence interval adjustment, for simple main effects are indicated by the following superscripts: difference from baseline value, † = $p<0.05$ (‡ = $p>0.05$ to $p<0.10$); and between groups ∗ = $p<0.05$ (⁑ = $p>0.05$ to $p<0.10$). η^2 effect size values of 0.01 - 0.05 = small, 0.06 - 0.13 = medium, and >0.14 = large.

Table S7: Corsi Block test results.

Variable	Group	N	Day 0 Pre	Day 0 Post	Day 30 Pre	Day 30 Post	Mean (SEM)	Effect	p-Level	η_p^2
Span Score	PL	*29*	6.54 ± 0.89	6.57 ± 0.67	6.53 ± 0.85	6.54 ± 0.91	6.55 ± 0.12	Group	0.998	0.000
	ASH	*30*	6.61 ± 0.71	6.64 ± 0.98	6.36 ± 0.67	6.57 ± 0.91	6.55 ± 0.12	Time	0.509	0.013
	Time	*59*	6.57 ± 0.79	6.61 ± 0.84	6.44 ± 0.76	6.56 ± 0.90	6.55 ± 0.08	G x T	0.673	0.009

Data are expressed as means ± standard deviations for the placebo (PL) and Ashwagandha (ASH) groups. Data were analyzed using a multivariate and univariate General Linear Model with repeated measures. P-levels, with partial ETA squared (η_p^2), are listed for between-subject group (G) and univariate within-subject (Greenhouse-Geisser) time (T), and group x time (G x T) effects. Multivariate Wilk's Lambda showed no significant time ($p = 0.592$, $\eta_p^2 = 0.034$), or group x time ($p = 0.754$, $\eta_p^2 = 0.021$), within-subject effects. Pairwise comparisons, with LSD confidence interval adjustment, for simple main effects are indicated by the following superscripts: difference from baseline value, † = $p<0.05$ (‡ = $p>0.05$ to $p<0.10$); and between groups ∗ = $p<0.05$ (⁑ = $p>0.05$ to $p<0.10$). η_p^2 effect size values of 0.01 - 0.05 = small, 0.06 - 0.13 = medium, and >0.14 = large.

Table S8: Stroop Color Word test results.

Variable	Group	N	Day 0 Pre	Day 0 Post	Day 30 Pre	Day 30 Post	Mean (SEM)	Effect	p-Level	η^2
Words Correct	PL	*29*	99.76 ± 0.58	99.25 ± 1.05 ‡	99.20 ± 1.38	99.25 ± 1.05	99.37 ± 0.14	Group	0.372	0.014
(%)	ASH	*30*	99.14 ± 1.56	99.22 ± 1.14	99.22 ± 1.89	99.17 ± 1.14	99.19 ± 0.14	Time	0.617	0.010
	Time	*59*	99.44 ± 1.21 ∗	99.24 ± 1.09	99.21 ± 1.65	99.21 ± 1.09	99.28 ± 0.10	G x T	0.413	0.016
Overall Reaction Time	PL	*29*	916.5 ± 144.0	860.3 ± 115.9 †	911.8 ± 174.3	876.6 ± 146.2 ‡	891.3 ± 21.1	Group	0.711	0.002
(ms)	ASH	*30*	918.3 ± 154.9	845.6 ± 74.1 †	906.1 ± 146.6	851.2 ± 108.1 †	880.3 ± 20.7	Time	0.000	0.124
	Time	*59*	917.4 ± 148.3	852.9 ± 96.4 †	908.9 ± 159.5	863.7 ± 127.8 †	885.8 ± 14.8	G x T	0.773	0.005
Correct Reaction Time	PL	*29*	913.2 ± 143.3	861.0 ± 115.8 †	913.3 ± 176.1	877.3 ± 145.8	891.2 ± 21.0	Group	0.692	0.003
(ms)	ASH	*30*	917.5 ± 152.5	846.1 ± 74.1 †	902.2 ± 144.2	852.0 ± 107.6 †	879.5 ± 20.7	Time	0.000	0.117
	Time	*59*	915.4 ± 146.8	853.4 ± 96.3 †	907.7 ± 159.4	864.5 ± 127.3 †	885.3 ± 14.7	G x T	0.751	0.005
Congruent Words Correct	PL	*29*	99.89 ± 0.62	98.97 ± 1.80 †	99.20 ± 1.70 ‡	98.74 ± 2.07 †	99.20 ± 0.16	Group	0.258	0.022
(%)	ASH	*30*	99.44 ± 1.54	99.33 ± 1.35	99.67 ± 1.02	99.33 ± 1.35	99.45 ± 0.15	Time	0.087	0.039
	Time	*59*	99.66 ± 1.19	99.15 ± 1.59 †	99.44 ± 1.40	99.04 ± 1.76 †	99.32 ± 0.11	G x T	0.200	0.027
Incongruent Words Correct	PL	*29*	99.64 ± 1.03	99.54 ± 1.17	99.20 ± 1.92	99.77 ± 0.86	99.54 ± 0.20	Group	0.033	0.078
(%)	ASH	*30*	98.85 ± 2.19	99.11 ± 1.74	98.78 ± 3.21	99.00 ± 1.99	98.94 ± 0.19	Time	0.633	0.009
	Time	*59*	99.24 ± 1.75 ⁑	99.32 ± 1.49	98.98 ± 2.64	99.38 ± 1.57 ⁑	99.24 ± 0.14 ∗	G x T	0.882	0.003
Congruent Words Overall Reaction Time	PL	*29*	893.5 ± 131.6	853.9 ± 120.4 †	867.9 ± 122.3	853.6 ± 137.1 †	867.2 ± 18.6	Group	0.428	0.011
(ms)	ASH	*30*	872.1 ± 94.9	820.3 ± 69.5 †	864.7 ± 128.0	828.3 ± 99.3 †	846.4 ± 18.3	Time	0.000	0.106
	Time	*59*	882.6 ± 113.9	836.8 ± 98.5 †	866.3 ± 124.1	840.7 ± 119.0 †	856.8 ± 13.1	G x T	0.605	0.010
Incongruent Words Overall Reaction Time	PL	*29*	938.2 ± 165.7	866.8 ± 119.4 †	955.7 ± 258.7	899.6 ± 161.8	915.1 ± 24.7	Group	0.981	0.000
(ms)	ASH	*30*	964.5 ± 236.7	871.0 ± 90.2 †	947.4 ± 180.1	874.1 ± 124.3 †	914.3 ± 24.3	Time	0.003	0.099
	Time	*59*	951.6 ± 203.6	868.9 ± 104.7 †	951.5 ± 220.3	886.6 ± 143.3 †	914.7 ± 17.3	G x T	0.660	0.007
Congruent Words Correct Reaction Time	PL	*29*	893.6 ± 131.6	855.1 ± 120.1 †	869.1 ± 123.6	855.1 ± 136.7 †	868.2 ± 18.7	Group	0.419	0.011
(ms)	ASH	*30*	872.4 ± 94.4	820.9 ± 69.9 †	865.3 ± 127.7	829.1 ± 98.8 †	846.9 ± 18.3	Time	0.000	0.104
	Time	*59*	882.8 ± 113.7	837.7 ± 98.5 †	867.2 ± 124.6	841.9 ± 118.6 †	857.6 ± 13.1	G x T	0.597	0.010
Incongruent Words Correct Reaction Time	PL	*29*	930.3 ± 164.0	866.8 ± 119.6	958.2 ± 264.9	899.4 ± 161.7	913.7 ± 28.4	Group	0.684	0.003
(ms)	ASH	*30*	1033.7 ± 529.3	871.5 ± 90.1 †	939.6 ± 173.4	875.1 ± 124.1 †	930.0 ± 27.9	Time	0.055	0.056
	Time	*59*	982.8 ± 394.7	869.2 ± 104.7 †	948.7 ± 221.3	887.1 ± 143.1 ‡	921.8 ± 19.9	G x T	0.323	0.019

Data are expressed as means ± standard deviations for the placebo (PL) and Ashwagandha (ASH) groups. Data were analyzed using a multivariate and univariate General Linear Model with repeated measures. P-levels, with partial ETA squared (η^2), are listed for between-subject group (G) and univariate within-subject (Greenhouse-Geisser) time (T), and group x time (G x T) effects. Multivariate Wilk's Lambda showed significant time ($p = 0.040$, $\eta^2 = 0.079$), but no group x time ($p = 0.514$, $\eta^2 = 0.051$), within-subject effects. Pairwise comparisons, with LSD confidence interval adjustment, for simple main effects are indicated by the following superscripts: difference from baseline value, † = p<0.05 (‡ = p>0.05 to p<0.10); and between groups ∗ = p<0.05 (⁑ = p>0.05 to p<0.10). η^2 effect size values of 0.01 - 0.05 = small, 0.06 - 0.13 = medium, and >0.14 = large.

Table S9: Profile of Mood States results.

Variable	Group	*N*	Day 0 Pre	Day 0 Post	Day 30 Pre	Day 30 Post	Mean (SEM)	Effect	p-Value	η_p^2
Tension	PL	*29*	8.93 ± 5.48	6.79 ± 6.18 †	7.79 ± 5.83	6.31 ± 5.53 †	7.46 ± 0.76	Group	0.804	0.001
	ASH	*30*	9.30 ± 3.72	7.33 ± 3.53 †	7.00 ± 4.19 †	5.13 ± 3.24 †	7.19 ± 0.75	Time	0.000	0.189
	Time	*59*	9.12 ± 4.63	7.07 ± 4.97 †	7.39 ± 5.04 †	5.71 ± 4.51 †	7.32 ± 0.53	G x T	0.297	0.021
Depression	PL	*29*	5.10 ± 6.80	3.97 ± 6.63 ‡	3.62 ± 4.87 ‡	2.14 ± 3.81 †	3.71 ± 0.83	Group	0.574	0.006
	ASH	*30*	4.23 ± 5.09	2.77 ± 4.80 †	2.97 ± 4.24	2.23 ± 3.46 †	3.05 ± 0.81	Time	0.000	0.120
	Time	*59*	4.66 ± 5.96	3.36 ± 5.75 †	3.29 ± 4.53 †	2.19 ± 3.60 †	3.38 ± 0.58	G x T	0.584	0.010
Anger	PL	*29*	4.79 ± 5.60	3.07 ± 5.72 †	3.31 ± 4.96 †	2.00 ± 3.82 †	3.29 ± 0.69	Group	0.391	0.013
	ASH	*30*	3.30 ± 3.19	2.70 ± 3.40	2.50 ± 4.34	1.30 ± 2.49 †	2.45 ± 0.68	Time	0.000	0.139
	Time	*59*	4.03 ± 4.56	2.88 ± 4.65 †	2.90 ± 4.63 †	1.64 ± 3.20 †	2.87 ± 0.49	G x T	0.617	0.009
Fatigue	PL	*29*	6.83 ± 4.50	5.59 ± 4.96 †	6.21 ± 4.69	4.83 ± 5.11 †	5.86 ± 0.83	Group	0.816	0.001
	ASH	*30*	7.87 ± 5.39	6.67 ± 4.80 †	5.33 ± 4.59 †	4.67 ± 4.54 †	6.13 ± 0.81	Time	0.000	0.200
	Time	*59*	7.36 ± 4.96	6.14 ± 4.87 †	5.76 ± 4.62 †	4.75 ± 4.79 †	6.00 ± 0.58	G x T	0.058	0.047
Confusion	PL	*29*	6.17 ± 2.71	6.03 ± 3.86	4.76 ± 2.21 †	4.66 ± 2.41 †	5.41 ± 0.49	Group	0.930	0.000
	ASH	*30*	6.50 ± 3.48	5.60 ± 3.76 ‡	5.17 ± 3.44 †	4.60 ± 2.79 †	5.47 ± 0.49	Time	0.000	0.145
	Time	*59*	6.34 ± 3.11	5.81 ± 3.78	4.97 ± 2.88 †	4.63 ± 2.59 †	5.44 ± 0.35	G x T	0.592	0.010
Vigor	PL	*29*	15.34 ± 6.39	14.10 ± 6.48 †	14.55 ± 6.16	13.48 ± 7.53 ‡	14.37 ± 1.04	Group	0.903	0.000
	ASH	*30*	15.70 ± 5.32	13.67 ± 5.61 †	14.40 ± 5.41	13.00 ± 6.28 †	14.19 ± 1.02	Time	0.004	0.098
	Time	*59*	15.53 ± 5.82	13.88 ± 6.01 †	14.47 ± 5.74 ‡	13.24 ± 6.86 †	14.28 ± 0.73	G x T	0.767	0.004
Total Mood Disturbance Score	PL	*29*	16.48 ± 25.13	11.34 ± 27.71 †	11.14 ± 21.27 †	6.45 ± 19.71 †	11.35 ± 3.52	Group	0.800	0.001
	ASH	*30*	15.50 ± 17.52	11.40 ± 16.07 †	8.57 ± 18.35 †	4.93 ± 15.39 †	10.10 ± 3.46	Time	0.000	0.188
	Time	*59*	15.98 ± 21.42	11.37 ± 22.35 †	9.83 ± 19.71 †	5.68 ± 17.51 †	10.73 ± 2.47	G x T	0.798	0.004

Data are expressed as means ± standard deviations for the placebo (PL) and Ashwagandha (ASH) groups. Data were analyzed using a multivariate and univariate General Linear Model with repeated measures. P-levels, with partial ETA squared (η_p^2), are listed for between-subject group (G) and univariate within-subject (Greenhouse-Geisser) time (T), and group x time (G x T) effects. Multivariate Wilk's Lambda showed significant time ($p<0.001$, $\eta_p^2 = 0.148$), but no group x time ($p = 0.148$, $\eta_p^2 = 0.047$), within-subject effects. Pairwise comparisons, with LSD confidence interval adjustment, for simple main effects are indicated by the following superscripts: difference from baseline value, † = p<0.05 (‡ = p>0.05 to p<0.10); and between groups ∗ = p<0.05 (⁑ = p>0.05 to p<0.10). η_p^2 effect size values of 0.01 - 0.05 = small, 0.06 - 0.13 = medium, and >0.14 = large.

Table S10: Cell Blood Count data.

Variable	Group	N	Day 0	Day 30	Mean (SEM)	Effect	p-Level	η^2
White Blood Cells	PL	*29*	5.89 ± 1.70	5.58 ± 1.47	5.73 ± 0.25	Group	0.134	0.039
(K/μL)	ASH	*30*	6.28 ± 1.32	6.27 ± 1.56	6.28 ± 0.25	Time	0.346	0.016
	Time	*59*	6.09 ± 1.52	5.93 ± 1.54 ⁑	6.00 ± 0.18	G x T	0.398	0.013
Red Blood Cells	PL	*29*	4.70 ± 0.83	4.70 ± 0.43	4.70 ± 0.10	Group	0.169	0.033
(M/μL)	ASH	*30*	4.88 ± 0.53	4.92 ± 0.50	4.90 ± 0.10	Time	0.696	0.003
	Time	*59*	4.79 ± 0.69	4.81 ± 0.48 ⁑	4.80 ± 0.07	G x T	0.773	0.001
Hemoglobin	PL	*29*	14.14 ± 1.47	13.86 ± 1.71	14.00 ± 0.33	Group	0.901	0.000
(g/dL)	ASH	*30*	14.21 ± 1.64	13.90 ± 2.93	14.06 ± 0.33	Time	0.239	0.024
	Time	*59*	14.18 ± 1.54	13.88 ± 2.39	14.03 ± 0.23	G x T	0.944	0.000
Hematocrit	PL	*29*	42.25 ± 3.94	40.97 ± 3.91 †	41.61 ± 0.76	Group	0.412	0.012
(%)	ASH	*30*	42.34 ± 4.57	42.64 ± 4.55	42.49 ± 0.75	Time	0.112	0.044
	Time	*59*	42.29 ± 4.24	41.82 ± 4.30	42.05 ± 0.53	G x T	0.011	0.108
Mean Corpuscular Volume	PL	*29*	87.88 ± 4.91	87.22 ± 5.29	87.55 ± 1.27	Group	0.341	0.016
(fL)	ASH	*30*	87.04 ± 5.96	84.65 ± 13.13	85.84 ± 1.25	Time	0.185	0.031
	Time	*59*	87.45 ± 5.44	85.91 ± 10.07	86.70 ± 0.89	G x T	0.452	0.010
Mean Corpuscular Hemoglobin	PL	*29*	29.41 ± 2.13	29.29 ± 2.30	29.35 ± 0.55	Group	0.518	0.007
(pg)	ASH	*30*	28.36 ± 5.46	29.33 ± 2.72	28.85 ± 0.54	Time	0.364	0.014
	Time	*59*	28.88 ± 4.17	29.31 ± 2.50	29.10 ± 0.39	G x T	0.241	0.024
Mean Corpuscular Hemoglobin Concentration	PL	*29*	33.44 ± 0.95	33.54 ± 1.08	33.49 ± 0.16	Group	0.728	0.002
(g/dL)	ASH	*30*	33.55 ± 0.83	33.58 ± 1.07	33.57 ± 0.15	Time	0.603	0.005
	Time	*59*	33.49 ± 0.89	33.56 ± 1.07	33.53 ± 0.11	G x T	0.782	0.001
Red Blood Cell Distribution Width	PL	*29*	12.48 ± 0.75	12.41 ± 0.84	12.44 ± 0.16	Group	0.333	0.016
(%)	ASH	*30*	12.65 ± 0.98	12.68 ± 0.96	12.67 ± 0.16	Time	0.650	0.004
	Time	*59*	12.57 ± 0.88	12.55 ± 0.91	12.55 ± 0.11	G x T	0.337	0.016
Neutrophils	PL	*29*	54.60 ± 8.11	53.60 ± 8.99	54.10 ± 1.40	Group	0.105	0.045
(%)	ASH	*30*	56.94 ± 8.23	57.73 ± 8.89	57.33 ± 1.38	Time	0.921	0.000
	Time	*59*	55.79 ± 8.19	55.70 ± 9.10 ⁑	55.72 ± 0.98	G x T	0.399	0.012
Lymphocytes	PL	*29*	33.94 ± 7.55	34.79 ± 8.11	34.36 ± 1.30	Group	0.052	0.065
(%)	ASH	*30*	31.66 ± 7.42	29.81 ± 9.02	30.74 ± 1.28	Time	0.629	0.004
	Time	*59*	32.78 ± 7.51	32.26 ± 8.87 ∗	32.55 ± 0.91 ⁑	G x T	0.193	0.030
Monocytes	PL	*29*	7.57 ± 1.55	7.74 ± 1.69	7.66 ± 0.30	Group	0.605	0.005
(%)	ASH	*30*	7.70 ± 2.02	8.06 ± 1.83	7.88 ± 0.30	Time	0.176	0.032
	Time	*59*	7.64 ± 1.79	7.91 ± 1.75	7.77 ± 0.21	G x T	0.624	0.004
Eosinophils	PL	*29*	2.93 ± 2.98	2.85 ± 2.94	2.89 ± 0.44	Group	0.695	0.003
(%)	ASH	*30*	2.60 ± 1.72	2.70 ± 1.88	2.65 ± 0.43	Time	0.935	0.000
	Time	*59*	2.76 ± 2.40	2.77 ± 2.43	2.77 ± 0.31	G x T	0.588	0.005
Basophils	PL	*29*	0.68 ± 0.31	0.75 ± 0.42	0.71 ± 0.06	Group	0.365	0.014
(%)	ASH	*30*	0.81 ± 0.31	0.77 ± 0.37	0.79 ± 0.06	Time	0.735	0.002
	Time	*59*	0.74 ± 0.32	0.76 ± 0.39	0.75 ± 0.04	G x T	0.203	0.028
Platelets	PL	*29*	261.5 ± 48.2	264.9 ± 59.1	263.2 ± 11.1	Group	0.917	0.000
(K/μL)	ASH	*30*	252.7 ± 67.8	270.4 ± 76.7 †	261.5 ± 10.9	Time	0.084	0.051
	Time	*59*	257.0 ± 58.7	267.7 ± 68.1 ‡	262.3 ± 7.8	G x T	0.238	0.024

Data are expressed as means ± standard deviations for the placebo (PL) and Ashwagandha (ASH) groups. Data were analyzed using a multivariate and univariate General Linear Model with repeated measures. P-levels, with partial ETA squared (η^2), are listed for between-subject group (G) and univariate within-subject (Greenhouse-Geisser) time (T), and group x time (G x T) effects. Multivariate Wilk's Lambda showed no significant time ($p = 0.214$, $\eta^2 = 0.302$), nor group x time ($p = 0.291$, $\eta^2 = 0.281$), within-subject effects. Pairwise comparisons, with LSD confidence interval adjustment, for simple main effects are indicated by the following superscripts: difference from baseline value, † = p<0.05 (‡ = p>0.05 to p<0.10); and between groups ∗ = p<0.05 (⁑ = p>0.05 to p<0.10). η^2 effect size values of 0.01 - 0.05 = small, 0.06 - 0.13 = medium, and >0.14 = large.

Table S11: Serum Metabolic Panel.

Variable	Group	N	Day 0	Day 30	Mean (SEM)	Effect	p-Level	η^2
Total Cholesterol	PL	*29*	171.2 ± 24.4	173.8 ± 27.4	172.5 ± 4.2	Group	0.330	0.017
(mg/dL)	ASH	*30*	162.9 ± 21.4	170.6 ± 23.4 ∗	166.7 ± 4.1	Time	0.024	0.086
	Time	*59*	167.0 ± 23.1	172.2 ± 25.3 †	169.6 ± 2.9	G x T	0.255	0.023
Triglycerides	PL	*29*	85.6 ± 36.4	81.5 ± 32.4	83.6 ± 5.6	Group	0.834	0.001
(mg/dL)	ASH	*30*	83.2 ± 31.4	80.7 ± 32.4	81.9 ± 5.5	Time	0.356	0.015
	Time	*59*	84.4 ± 33.7	81.1 ± 32.1	82.7 ± 3.9	G x T	0.821	0.001
HDL Cholesterol	PL	*29*	57.3 ± 12.3	57.4 ± 15.4	57.3 ± 2.3	Group	0.193	0.030
(mg/dL)	ASH	*30*	50.6 ± 10.2	55.5 ± 13.8 ∗	53.1 ± 2.3	Time	0.015	0.099
	Time	*59*	53.9 ± 11.6	56.5 ± 14.5 †∗	55.2 ± 1.6	G x T	0.023	0.088
LDL Cholesterol	PL	*29*	96.4 ± 18.4	99.3 ± 24.5	97.8 ± 3.6	Group	0.763	0.002
(mg/dL)	ASH	*30*	94.7 ± 19.0	97.9 ± 19.5	96.3 ± 3.5	Time	0.105	0.045
	Time	*59*	95.5 ± 18.6	98.6 ± 21.9	97.1 ± 2.5	G x T	0.951	0.000
Non-HDL Cholesterol	PL	*29*	114.0 ± 21.7	116.4 ± 26.9	115.2 ± 4.1	Group	0.791	0.001
(mg/dL)	ASH	*30*	112.3 ± 21.8	115.1 ± 22.8	113.7 ± 4.0	Time	0.192	0.030
	Time	*59*	113.1 ± 21.6	115.7 ± 24.7	114.4 ± 2.9	G x T	0.927	0.000
VLDL Cholesterol	PL	*29*	17.60 ± 5.03	17.14 ± 4.30	17.37 ± 0.79	Group	0.994	0.000
(mg/dL)	ASH	*30*	17.52 ± 4.30	17.20 ± 4.55	17.36 ± 0.77	Time	0.369	0.014
	Time	*59*	17.56 ± 4.63	17.17 ± 4.39	17.36 ± 0.55	G x T	0.872	0.000
LDL-HDL Ratio	PL	*29*	2.63 ± 3.82	1.88 ± 0.75	2.25 ± 0.28	Group	0.413	0.012
	ASH	*30*	1.97 ± 0.58	1.89 ± 0.69	1.93 ± 0.27	Time	0.226	0.026
	Time	*59*	2.29 ± 2.71	1.88 ± 0.72	2.09 ± 0.20	G x T	0.332	0.017
Total Cholesterol/HDL Ratio	PL	*29*	3.12 ± 0.64	3.21 ± 0.86	3.16 ± 0.25	Group	0.106	0.045
	ASH	*30*	4.27 ± 3.53	3.22 ± 0.83 ∗	3.75 ± 0.25	Time	0.170	0.033
	Time	*59*	3.70 ± 2.60	3.22 ± 0.84 ⁑	3.45 ± 0.18	G x T	0.107	0.045
Glucose	PL	*29*	90.71 ± 5.86	92.52 ± 15.03	91.61 ± 1.42	Group	0.257	0.022
(mg/dL)	ASH	*30*	94.37 ± 6.77	93.43 ± 7.56	93.90 ± 1.40	Time	0.766	0.002
	Time	*59*	92.57 ± 6.55	92.98 ± 11.75 ∗	92.76 ± 1.00	G x T	0.352	0.015
Blood Urea Nitrogen	PL	*29*	13.39 ± 3.68	14.45 ± 3.62 ‡	13.92 ± 0.63	Group	0.584	0.005
(mg/dL)	ASH	*30*	13.00 ± 3.70	13.87 ± 4.04	13.43 ± 0.62	Time	0.032	0.078
	Time	*59*	13.19 ± 3.66	14.15 ± 3.82 †	13.68 ± 0.44	G x T	0.824	0.001
Creatinine	PL	*29*	0.93 ± 0.18	0.92 ± 0.20	0.92 ± 0.04	Group	0.766	0.002
(mg/dL)	ASH	*30*	0.93 ± 0.19	0.95 ± 0.20	0.94 ± 0.03	Time	0.712	0.002
	Time	*59*	0.93 ± 0.19	0.93 ± 0.20	0.93 ± 0.02	G x T	0.091	0.049
Estimated Glomerular Filtration Rate	PL	*29*	102.7 ± 15.6	105.1 ± 17.7	103.9 ± 3.1	Group	0.536	0.007
(ml/min/1.73)	ASH	*30*	102.7 ± 17.3	99.7 ± 18.1 ⁑	101.2 ± 3.0	Time	0.788	0.001
	Time	*59*	102.7 ± 16.3	102.4 ± 17.9	102.6 ± 2.2	G x T	0.025	0.085
BUN-Creatinine Ratio	PL	*29*	14.40 ± 4.32	16.14 ± 4.10 ⁑	15.27 ± 0.62	Group	0.378	0.014
	ASH	*30*	14.27 ± 3.10	14.73 ± 3.60	14.50 ± 0.61	Time	0.026	0.084
	Time	*59*	14.33 ± 3.72	15.42 ± 3.89 †	14.88 ± 0.43	G x T	0.191	0.030
Sodium	PL	*29*	141.0 ± 2.6	141.2 ± 1.8	141.1 ± 0.3	Group	0.584	0.005
(meq/L)	ASH	*30*	140.6 ± 1.8	141.2 ± 2.1	140.9 ± 0.3	Time	0.273	0.021
	Time	*59*	140.8 ± 2.2	141.2 ± 1.9	141.0 ± 0.2	G x T	0.514	0.008
Potassium	PL	*29*	4.48 ± 0.61	4.44 ± 0.41	4.46 ± 0.07	Group	0.881	0.000
(meq/L)	ASH	*30*	4.35 ± 0.65	4.55 ± 0.36	4.45 ± 0.07	Time	0.371	0.014
	Time	*59*	4.41 ± 0.63	4.50 ± 0.38	4.46 ± 0.05	G x T	0.197	0.029
Chloride	PL	*29*	103.1 ± 2.1	103.0 ± 2.0	103.0 ± 0.3	Group	0.659	0.003
(meq/L)	ASH	*30*	103.3 ± 2.0	103.2 ± 2.2	103.2 ± 0.3	Time	0.826	0.001
	Time	*59*	103.2 ± 2.0	103.1 ± 2.1	103.1 ± 0.2	G x T	0.997	0.000
Carbon Dioxide	PL	*29*	25.36 ± 2.18	26.00 ± 2.00 ⁑	25.68 ± 0.31	Group	0.708	0.002
(meq/L)	ASH	*30*	25.40 ± 1.94	25.63 ± 1.47	25.52 ± 0.30	Time	0.081	0.052
	Time	*59*	25.38 ± 2.05	25.81 ± 1.75 ‡	25.60 ± 0.22	G x T	0.412	0.012
Calcium	PL	*29*	9.86 ± 0.39	9.88 ± 0.36	9.87 ± 0.05	Group	0.252	0.023
(mg/dL)	ASH	*30*	9.88 ± 0.27	10.04 ± 0.37 ∗	9.96 ± 0.05	Time	0.082	0.052
	Time	*59*	9.87 ± 0.33	9.96 ± 0.37 ⁑	9.91 ± 0.04	G x T	0.152	0.036
Total Proteins	PL	*29*	7.27 ± 0.40	7.21 ± 0.43	7.24 ± 0.06	Group	0.541	0.007
(g/dL)	ASH	*30*	7.21 ± 0.39	7.37 ± 0.31 ∗	7.29 ± 0.06	Time	0.254	0.023
	Time	*59*	7.24 ± 0.39	7.29 ± 0.38 ‡⁑	7.27 ± 0.04	G x T	0.019	0.093
Albumin	PL	*29*	4.76 ± 0.29	4.74 ± 0.29	4.75 ± 0.05	Group	0.376	0.014
(g/dL)	ASH	*30*	4.76 ± 0.30	4.86 ± 0.29 ∗	4.81 ± 0.05	Time	0.205	0.028
	Time	*59*	4.76 ± 0.29	4.80 ± 0.29	4.78 ± 0.04	G x T	0.045	0.068
Globulin	PL	*29*	2.50 ± 0.32	2.47 ± 0.32	2.48 ± 0.25	Group	0.353	0.015
(g/dL)	ASH	*30*	3.12 ± 3.71	2.51 ± 0.28	2.82 ± 0.25	Time	0.365	0.014
	Time	*59*	2.81 ± 2.65	2.49 ± 0.30	2.65 ± 0.18	G x T	0.403	0.012
Albumin-Globulin Ratio	PL	*29*	1.95 ± 0.32	1.95 ± 0.29	1.95 ± 0.05	Group	0.752	0.002
	ASH	*30*	1.98 ± 0.35	1.96 ± 0.29	1.97 ± 0.05	Time	0.699	0.003
	Time	*59*	1.97 ± 0.33	1.96 ± 0.29	1.96 ± 0.04	G x T	0.740	0.002
Bilirubin	PL	*29*	0.62 ± 0.42	0.65 ± 0.39	0.63 ± 0.06	Group	0.078	0.053
(mg/dL)	ASH	*30*	0.52 ± 0.19	0.47 ± 0.18	0.50 ± 0.05	Time	0.848	0.001
	Time	*59*	0.57 ± 0.32	0.56 ± 0.32 ∗	0.56 ± 0.04 ∗	G x T	0.115	0.043
Alkaline Phosphatase	PL	*29*	72.90 ± 26.76	73.59 ± 26.52	73.24 ± 4.50	Group	0.501	0.008
(U/L)	ASH	*30*	74.33 ± 22.09	80.70 ± 22.82 ∗	77.52 ± 4.43	Time	0.002	0.155
	Time	*59*	73.63 ± 24.30	77.20 ± 24.76 †	75.38 ± 3.16	G x T	0.012	0.106
Aspartate Aminotransferase	PL	*29*	21.24 ± 4.73	21.69 ± 6.71	21.46 ± 1.45	Group	0.377	0.014
(U/L)	ASH	*30*	23.33 ± 12.18	23.20 ± 9.02	23.27 ± 1.42	Time	0.872	0.000
	Time	*59*	22.30 ± 9.28	22.46 ± 7.94	22.37 ± 1.01	G x T	0.768	0.002
Alanine Aminotransferase	PL	*29*	16.96 ± 6.56	20.38 ± 9.58	18.67 ± 3.15	Group	0.270	0.021
(U/L)	ASH	*30*	25.23 ± 37.23	21.60 ± 10.94	23.42 ± 3.10	Time	0.971	0.000
	Time	*59*	21.17 ± 27.04	21.00 ± 10.23	21.04 ± 2.21	G x T	0.236	0.024

Data are expressed as means ± standard deviations for the placebo (PL) and Ashwagandha (ASH) groups. Data were analyzed using a multivariate and univariate General Linear Model with repeated measures. P-levels, with partial ETA squared (η^2), are listed for between-subject group (G) and univariate within-subject (Greenhouse-Geisser) time (T), and group x time (G x T) effects. Multivariate Wilk's Lambda showed significant time ($p = 0.040$, $\eta^2 = 0.575$), but not group x time ($p = 0.102$, $\eta^2 = 0.530$), within-subject effects. Pairwise comparisons, with LSD confidence interval adjustment, for simple main effects are indicated by the following superscripts: difference from baseline value, † = p<0.05 (‡ = p>0.05 to p<0.10); and between groups ∗ = p<0.05 (⁑ = p>0.05 to p<0.10). η^2 effect size values of 0.01 - 0.05 = small, 0.06 - 0.13 = medium, and >0.14 = large.

Table S12: Frequency and severity of side effects.

Frequency

Symptom	Day	Group	None	1-2x's per week	5-6x's per week	7-8x's per week	3-4x's per week	>9x's per week	χ^2
Dizziness	Day 1 Pre	PLA	24	4	1	0	0	0	0.553
		ASH	24	5	0	0	0	1	
		Total	48	9	1	0	0	1	
	Day 1 Post	PLA	25	3	1	0	0	0	0.475
		ASH	24	5	0	0	0	1	
		Total	49	8	1	0	0	1	
	Day 30 Pre	PLA	24	4	1	0	0	0	0.351
		ASH	28	1	1	0	0	0	
		Total	52	5	2	0	0	0	
	Day 30 Post	PLA	25	4	0	0	0	0	0.422
		ASH	27	2	1	0	0	0	
		Total	52	6	1	0	0	0	
Headache	Day 1 Pre	PLA	10	16	3	0	0	0	0.190
		ASH	17	9	3	1	0	0	
		Total	27	25	6	1	0	0	
	Day 1 Post	PLA	18	8	3	0	0	0	0.531
		ASH	19	5	5	1	0	0	
		Total	37	13	8	1	0	0	
	Day 30 Pre	PLA	15	13	1	0	0	0	0.365
		ASH	15	10	4	0	1	0	
		Total	30	23	5	0	1	0	
	Day 30 Post	PLA	20	8	1	0	0	0	0.518
		ASH	20	6	3	0	1	0	
		Total	40	14	4	0	1	0	
Tachycardia	Day 1 Pre	PLA	19	9	1	0	0	0	0.140
		ASH	22	4	4	0	0	0	
		Total	41	13	5	0	0	0	
	Day 1 Post	PLA	25	3	1	0	0	0	0.241
		ASH	22	3	5	0	0	0	
		Total	47	6	6	0	0	0	
	Day 30 Pre	PLA	23	5	0	1	0	0	0.571
		ASH	24	5	1	0	0	0	
		Total	47	10	1	1	0	0	
	Day 30 Post	PLA	25	3	0	1	0	0	0.589
		ASH	27	3	0	0	0	0	
		Total	52	6	0	1	0	0	
Heart Palpitations	Day 1 Pre	PLA	29	0	0	0	0	0	-
		ASH	30	0	0	0	0	0	
		Total	59	0	0	0	0	0	
	Day 1 Post	PLA	29	0	0	0	0	0	-
		ASH	30	0	0	0	0	0	
		Total	59	0	0	0	0	0	
	Day 30 Pre	PLA	29	0	0	0	0	0	-
		ASH	30	0	0	0	0	0	
		Total	59	0	0	0	0	0	
	Day 30 Post	PLA	29	0	0	0	0	0	-
		ASH	30	0	0	0	0	0	
		Total	59	0	0	0	0	0	
Dyspnea	Day 1 Pre	PLA	24	5	0	0	0	0	0.417
		ASH	27	3	0	0	0	0	
		Total	51	8	0	0	0	0	
	Day 1 Post	PLA	27	2	0	0	0	0	0.972
		ASH	28	2	0	0	0	0	
		Total	55	4	0	0	0	0	
	Day 30 Pre	PLA	26	3	0	0	0	0	0.723
		ASH	26	4	0	0	0	0	
		Total	52	7	0	0	0	0	
	Day 30 Post	PLA	28	1	0	0	0	0	0.574
		ASH	28	2	0	0	0	0	
		Total	56	3	0	0	0	0	
Nervousness	Day 1 Pre	PLA	11	12	3	3	0	0	0.491
		ASH	10	10	6	2	2	0	
		Total	21	22	9	5	2	0	
	Day 1 Post	PLA	20	5	2	2	0	0	0.359
		ASH	15	7	6	1	1	0	
		Total	35	12	8	3	1	0	
	Day 30 Pre	PLA	16	7	5	0	1	0	0.379
		ASH	17	6	4	3	0	0	
		Total	33	13	9	3	1	0	
	Day 30 Post	PLA	21	5	2	0	1	0	0.436
		ASH	20	4	4	2	0	0	
		Total	41	9	6	2	1	0	
Blurred Vision	Day 1 Pre	PLA	25	3	1	0	0	0	0.194
		ASH	29	0	1	0	0	0	
		Total	54	3	2	0	0	0	
	Day 1 Post	PLA	26	2	1	0	0	0	0.823
		ASH	28	1	1	0	0	0	
		Total	54	3	2	0	0	0	
	Day 30 Pre	PLA	24	3	2	0	0	0	0.998
		ASH	25	3	2	0	0	0	
		Total	49	6	4	0	0	0	
	Day 30 Post	PLA	25	3	1	0	0	0	0.498
		ASH	27	1	2	0	0	0	
		Total	52	4	3	0	0	0	
Other	Day 1 Pre	PLA	29	0	0	0	0	0	-
		ASH	30	0	0	0	0	0	
		Total	59	0	0	0	0	0	
	Day 1 Post	PLA	28	0	1	0	0	0	0.305
		ASH	30	0	0	0	0	0	
		Total	58	0	1	0	0	0	
	Day 30 Pre	PLA	29	0	0	0	0	0	-
		ASH	30	0	0	0	0	0	
		Total	59	0	0	0	0	0	
	Day 30 Post	PLA	29	0	0	0	0	0	-
		ASH	30	0	0	0	0	0	
		Total	59	0	0	0	0	0	

Severity

Symptom	Day	Group	None	Minimal	Slight	Moderate	Severe	Very Severe	χ^2
Dizziness	Day 1 Pre	PLA	24	2	2	1	0	0	0.941
		ASH	26	2	1	1	0	0	
		Total	50	4	3	2	0	0	
	Day 1 Post	PLA	25	2	2	0	0	0	0.361
		ASH	26	3	0	1	0	0	
		Total	51	5	2	1	0	0	
	Day 30 Pre	PLA	24	2	3	0	0	0	0.288
		ASH	28	0	2	0	0	0	
		Total	52	2	5	0	0	0	
	Day 30 Post	PLA	25	2	2	0	0	0	0.821
		ASH	27	2	1	0	0	0	
		Total	52	4	3	0	0	0	
Headache	Day 1 Pre	PLA	10	13	3	3	0	0	0.057
		ASH	18	4	5	3	0	0	
		Total	28	17	8	6	0	0	
	Day 1 Post	PLA	19	7	2	1	0	0	0.609
		ASH	20	4	4	2	0	0	
		Total	39	11	6	3	0	0	
	Day 30 Pre	PLA	15	11	2	1	0	0	0.230
		ASH	13	7	8	1	1	0	
		Total	28	18	10	2	1	0	
	Day 30 Post	PLA	20	5	3	1	0	0	0.730
		ASH	18	4	5	2	1	0	
		Total	38	9	8	3	1	0	
Tachycardia	Day 1 Pre	PLA	19	7	2	1	0	0	0.757
		ASH	23	4	2	1	0	0	
		Total	42	11	4	2	0	0	
	Day 1 Post	PLA	24	2	2	1	0	0	0.817
		ASH	22	3	4	1	0	0	
		Total	46	5	6	2	0	0	
	Day 30 Pre	PLA	23	5	1	0	0	0	0.799
		ASH	24	4	2	0	0	0	
		Total	47	9	3	0	0	0	
	Day 30 Post	PLA	24	4	1	0	0	0	0.902
		ASH	26	3	1	0	0	0	
		Total	50	7	2	0	0	0	
Heart Palpitations	Day 1 Pre	PLA	29	0	0	0	0	0	-
		ASH	30	0	0	0	0	0	
		Total	59	0	0	0	0	0	
	Day 1 Post	PLA	29	0	0	0	0	0	-
		ASH	30	0	0	0	0	0	
		Total	59	0	0	0	0	0	
	Day 30 Pre	PLA	29	0	0	0	0	0	0.321
		ASH	29	1	0	0	0	0	
		Total	58	1	0	0	0	0	
	Day 30 Post	PLA	29	0	0	0	0	0	0.321
		ASH	29	1	0	0	0	0	
		Total	58	1	0	0	0	0	
Dyspnea	Day 1 Pre	PLA	25	3	1	0	0	0	0.564
		ASH	26	4	0	0	0	0	
		Total	51	7	1	0	0	0	
	Day 1 Post	PLA	28	1	0	0	0	0	0.357
		ASH	26	3	0	1	0	0	
		Total	54	4	0	1	0	0	
	Day 30 Pre	PLA	26	3	0	0	0	0	0.965
		ASH	27	3	0	0	0	0	
		Total	53	6	0	0	0	0	
	Day 30 Post	PLA	28	1	0	0	0	0	0.574
		ASH	28	2	0	0	0	0	
		Total	56	3	0	0	0	0	
Nervousness	Day 1 Pre	PLA	11	10	5	3	0	0	0.606
		ASH	13	6	8	3	0	0	
		Total	24	16	13	6	0	0	
	Day 1 Post	PLA	21	2	4	2	0	0	0.188
		ASH	15	8	4	3	0	0	
		Total	36	10	8	5	0	0	
	Day 30 Pre	PLA	17	5	4	3	0	0	0.652
		ASH	19	2	5	4	0	0	
		Total	36	7	9	7	0	0	
	Day 30 Post	PLA	22	2	4	1	0	0	0.598
		ASH	20	2	4	4	0	0	
		Total	42	4	8	5	0	0	
Blurred Vision	Day 1 Pre	PLA	25	3	0	1	0	0	0.320
		ASH	29	1	0	0	0	0	
		Total	54	4	0	1	0	0	
	Day 1 Post	PLA	26	2	1	0	0	0	0.477
		ASH	29	1	0	0	0	0	
		Total	55	3	1	0	0	0	
	Day 30 Pre	PLA	25	3	0	1	0	0	0.547
		ASH	25	4	1	0	0	0	
		Total	50	7	1	1	0	0	
	Day 30 Post	PLA	24	4	1	0	0	0	0.662
		ASH	27	2	1	0	0	0	
		Total	51	6	2	0	0	0	
Other	Day 1 Pre	PLA	29	0	0	0	0	0	-
		ASH	30	0	0	0	0	0	
		Total	59	0	0	0	0	0	
	Day 1 Post	PLA	28	0	1	0	0	0	0.305
		ASH	30	0	0	0	0	0	
		Total	58	0	1	0	0	0	
	Day 30 Pre	PLA	29	0	0	0	0	0	-
		ASH	30	0	0	0	0	0	
		Total	59	0	0	0	0	0	
	Day 30 Post	PLA	29	0	0	0	0	0	-
		ASH	30	0	0	0	0	0	
		Total	59	0	0	0	0	0	

Data are presented as frequencies or the placebo (PLA) and Ashwaganda (ASH) groups. Statistical significance is detailed by chi-squared analysis; $p \leq 0.05$ considered significant. Totals are included but, separate from analysis.
